# Jianpi Huayu Prescription Prevents Atherosclerosis by Improving Inflammation and Reshaping the Intestinal Microbiota in ApoE^−/−^ Mice

**DOI:** 10.1007/s12013-024-01341-6

**Published:** 2024-08-22

**Authors:** Hao-Ran Zhao, Qin-Chi Xian, Xuan-Ming Zhang, Xiao-Yu Ma, Fu-Qiao Wang, Rui-Si Wang, Zhi-Jie Liu, Zhi-Gang Zhang

**Affiliations:** 1 First Affiliated Hospital of Shihezi University, Shihezi, 832000 China; 2 College of pharmacy, Shihezi University, Shihezi, 832000 China

**Keywords:** Jianpi Huayu Prescription, Atherosclerosis, Inflammation, Intestinal flora, TLR4/MyD88/MAPK signal path

## Abstract

This study established an LPS-induced RAW264.7 macrophage inflammatory injury model and an AS mouse vulnerable plaque model to observe the effect of JPHYP on macrophage inflammation, plaque formation, blood lipids, inflammation levels, intestinal flora and the influence of TLR4/MyD88/MAPK pathway, and explore the anti-AS effect and molecular mechanism of JPHYP, and detected 16S rRNA of mice intestinal microbes. The difference of intestinal flora in different groups of mice was compared to further explore the intervention effect of JPHYP and clarify the molecular biological mechanism of JPHYP in preventing and treating AS by regulating TLR4/MyD88/MAPK inflammatory signaling pathway and improving intestinal flora.

## Introduction

The “China Cardiovascular Health and Disease Report 2023” contains statistics that show how national lifestyle changes and social economic development have affected the prevalence of cardiovascular disease and its risk factors, which has a growing impact on citizens’ health [1]. The primary pathological underpinning of cardiovascular disease, atherosclerosis (AS), is also a major factor in the rising incidence of peripheral vascular illnesses such cerebral infarction and coronary heart disease. Every year, illnesses linked to AS claim the lives of almost 20 million individuals worldwide [2]. As a result, preventing AS is a crucial component in lowering the incidence of cardiovascular illnesses. For the sake of everyone’s health, prompt prevention and treatment of AS are crucial from a clinical and practical standpoint.

An inflammatory vascular disease that is chronic is AS. Foam cell production, vascular endothelial damage, inflammatory cell infiltration, etc. are the key pathophysiological factors [3]. One of the most prevalent and typical mechanisms in AS is inflammation. It serves as the common foundation for pathological and physiological alterations in the onset and progression of AS. There is an ongoing inflammatory response that interacts with adhesion molecules and inflammatory cytokines from the beginning to the end of AS. Preventing and treating AS requires reducing or eliminating inflammation because it is linked to different inflammatory cells, including inflammatory mediators [4–6]. A growing number of studies on the role of intestinal flora in lowering inflammation and anti-AS have been conducted in recent years. According to Yoshida et al. [7], Bacteroides vulgaris and Bacteroides doveri, which can lessen intestinal and systemic inflammation, were given intragastrically to the mouse AS model. inflammatory reaction, delaying the development of AS. According to Tong et al. [8], intestinal microbiota-produced anti-inflammatory metabolites and the regulation of the NLRP3 inflammasome pathway by whole wheat highland barley may reduce inflammation and the development of AS plaques. Numerous investigations have demonstrated a correlation between increased levels of circulating intestinal microbiota and inflammatory cytokines and the onset and progression of AS [9].

Among the primary signaling pathways in the development of AS, the Toll-like receptor 4 (TLR4)/myeloid differentiation factor 88 (MyD88)/mitogen-activated protein kinase (MAPK) pathway is a crucial mechanism in controlling inflammation. Regulation is impacted by a variety of cell processes, including differentiation, transformation, death, and proliferation [10]. Furthermore, a significant amount of lipopolysaccharide (LPS) is generated when there is an imbalance of Gram-negative bacteria in the intestine. LPS is an essential inflammatory stimulant that plays a pivotal role in the development of numerous inflammatory illnesses, including AS. It is a constituent of the outer layer of the cell wall of intestine negative bacteria.

It can break down the intestinal wall barrier, allowing bacteria to seep through. It then attaches itself to the TLR4 receptor on macrophages’ surface and activates MAPK, which causes the release of several inflammatory proteins by way of the MyD88 pathway, thereby starting an inflammatory response [11]. Thus, the goal of delaying the onset and progression of AS can be achieved by selectively inhibiting this signaling pathway, which can also decrease the infiltration of plaque cells, regulate intestinal flora, and inhibit the inflammatory response. It can be a crucial method for reversing AS.

The three main treatments for AS now available are medication therapy, coronary artery bypass grafting (CABG), and coronary artery intervention (PCI). Statins are the most widely used anti-AS medications in clinical practice, however they are only 30% effective in reducing the incidence on their own. rate and is followed by the development of unfavorable renal and liver reactions [12]. The range of biological activity and the variety of chemical components found in traditional Chinese medicine have made it theoretically feasible to prevent and treat AS effectively. With little adverse effects, it can cure AS in several linkages and targets. Consequently, the use of traditional Chinese medicine for the prevention and treatment of AS has emerged as a major area of scientific interest in recent years.

Our research team proposed that “spleen deficiency” is the basic pathogenesis of AS and that “phlegm and blood stasis” is the key factor in pathological changes based on the climatic characteristics of Xinjiang and the dietary habits of the residents. These findings establish that phlegm and spleen deficiency are the key factors in pathological changes. Blood stasis can be treated by stimulating the spleen, replenishing qi, clearing the phlegm, and removing blood stasis when it mixes with other conditions [13], In clinical practice, the traditional TCM prescription Sijunzi Decoction is combined with *Gynostemma Pentaphylla*, *Trichosanthes*, *Safflower*, etc. to create the Jianpi Huayu Prescription (JPHYP). Phlegm is cleared, blood stasis is removed, and the spleen is strengthened and qi is replenished.

According to the research team’s preliminary clinical investigations, JPHYP can lessen plaque area and help patients with cervical AS’s blood lipid metabolism issues and clinical symptoms [14]; Prior studies in network pharmacology have discovered a connection between the TLR4 signaling pathway and JPHYP’s management of AS [15]; It can lower blood cholesterol levels, prevent inflammatory responses, alleviate vascular plaque, and have anti-AS properties, according to animal studies. Nevertheless, research on the mechanism by which JPHYP treats AS is still lacking. It is uncertain how JPHYP affects the TLR4/MyD88/MAPK signaling pathway.

In order to determine if JPHYP improves AS by influencing the TLR4/MyD88/MAPK signaling pathway and thus lowering inflammation, we investigated JPHYP in vitro and in mouse aorta. It seeks to offer fresh perspectives on the use of traditional Chinese medicine ingredients to prevent and cure AS, as well as to offer a theoretical foundation and empirical evidence for the therapeutic use of these ingredients.

## Methods

### Medicine

JPHYP (20180870, The First Affiliated Hospital of Shihezi University) was prepared by double-distilling *Codonopsis pilosula* and *Stir-fried Atractylodes with bran*, *Poria*, *Tangerine peel*, *Salvia*, and other medicinal materials. The solution was concentrated to each milliliter by rotary evaporation, and 2.8 g of crude drug was obtained. The sample was freeze-dried at low temperature and vacuum to a constant weight and accurately weighed for later use. Atorvastatin (20 mg/tablet, EL2614) from Pfizer, Inc., was also used. Distilled water (500 mL/bottle) was purchased from Xinjiang Hua Shidan Pharmaceutical Co., Ltd.

### Ultra-high Performance Liquid Chromatography-high Resolution Mass Spectrometry Analysis

Studies were conducted using a Waters H-Class ultra-high performance liquid chromatograph (Waters Technologies Co., Ltd.) and an AB Sciex Triple TOF® 4600 high-resolution mass spectrometer (SCIEX Corporation).

Chromatographic conditions: Chromatographic column: Agilent InfinityLab Poroshell AQ-C18 (2.1 × 100 mm, 2.7 µm); column temperature: 30 °C; flow rate: 0.3 mL/min; injection volume: 2 µl; detection wavelength: 254 nm, 190~400 nm; mobile phase ratio: phase A acetonitrile, phase B 0.1% formic acid aqueous solution (Table 1).

**Table 1:** Mobile phase gradient

Time (min)	A%	B%
0~3	0	100
3~7	0~6	100~94
7~15	6~13	94~87
15~30	13~25	87~75
30~35	25~60	75~40
35~43	60~95	40~5
43~47	95	5
47~47.1	95~0	5~100
47.1~50	0	95

Mass spectrometry conditions: Mass spectrometry detection mode: ESI-Negative/Positive ion mode (Table 2).

**Table 2:** Mass parameters (Sciex Triple TOF 4600 LC-MS)

MS	Parameter value	MS/MS	Parameter value
TOF mass range	50~1700	MS/MS mass range	50~1250
Ion Source Gas 1 (psi)	50	Declustering Potential (V)	100
Ion Source Gas 2 (psi)	50	Collision Energy (eV)	±40
Curtain Gas (psi)	35	Collision Energy Spread (eV)	20
Ion Spray Voltage Floating (V)	-4500/5000	Ion Release Delay (ms)	30
Ion Source Temperature (°C)	500	Ion Release Width (ms)	15
Declustering Potential (V)	100		
Collision Energy (eV)	10		

### Cell Model Establishment

RAW264.7 macrophages (obtained from The First Affiliated Hospital of Shihezi University) were incubated with LPS (1 µg/mL) for 24 h to establish an inflammatory model for further experiments.

### Preparation of Medicated Serum

60 SD rats are randomly divided into 12 of the Control group, the Atorvastatin group, the JPHYP-L group, the JPHYP-M group, and the JPHYP-H group. Calculate the daily daily dose of adults based on JPHYP’s clinical use dose, and then calculate the dose of 18 g·kg^−1^ based on the “Adult-Rat Surface Conversion Form”. Therefore Dose and high doses are set to 9 g·kg^−1^, 36 g·kg^−1^. Atorvastatin adult (60 kg) takes the Atropine 20 mg daily. According to the ratio of human and rats, the ratio of rats can be converted to 2 mg·kg^−1^ Atropine. The control group (equal amounts of distilled water, i.g). After 1 week of intragastric administration, blood was taken from the abdominal aorta. The collected blood was allowed to stand for 2 h, followed by centrifugation at 3000 r·min^−1^ for 10 min. The serum was filtered through a 0.22 μm filter membrane and inactivated at 56 °C before being stored at −80 °C for future use.

### Cells Culture and Treatments

RAW264.7 macrophages were cultured in a 5% CO2 incubator at 37 °C, and high glucose medium (DMEM, catalog number WHB823D211, Punosai, China) and 10% fetal bovine serum (catalog number F90701, UE, China) were added to the culture flask, and 1% penicillin/streptomycin (catalog number P1400, Solarbio, China).

Following the removal of the therapeutic serum, dilute the serum to concentrations of 10%, 20%, 30%, 40%, 50%, 60%, 70%, 80%, or 90%; then, take RAW264.7 macrophages with various growth statuses. The 96-hole plate is inoculated with a density of 1.2*10^5 ^ml^−1^ per hole, and allowed to grow overnight. The cells are then examined under a microscope, and once the density of each hole has grown to around 80%, the group adds a different concentration of the medicinal serum 100 µL for each of the three settings. 3~5 compound holes, add 10 µL of CCK-8 working solution per hole after 24 hours of incubation, measure the cell OD value at 450 nm, ascertain the ideal serum dilution concentration for each group, and proceed to the next experiment.

Determine the optimal dilution factor. RAW264.7 macrophage culture medium was added with 1 ug/mL LPS (catalog number IL2020, Solarbio, China) and cultured for 24 h to form an inflammatory cell model. Serum containing JPHYP was added to the cell culture dish at a dose of 60% and cultured for 24 h. TAK-242 is a TLR4-specific blocker (catalog number CM00939, Wuhan Proteintech, China) that inhibits TLR4 at 10 nmol/L.

In order to detect the effect of JPHYP on cellular inflammation, we performed CLSM to detect the ability of JPHYP to inhibit ROS generation in RAW264.7 macrophages, and ELISA to detect the inflammatory factors TNF-α, IL-1β, IL-6 and MCP-1 in the cells (catalog numbers E-EL-M3063, E-EL-M0037c, E-EL-M0044c, E-EL-M3001) levels.

### Animals

A total of 60 male apolipoprotein E (ApoE^−/−^) gene knockout mice aged 8 weeks and 12 male C57BL/6J mice of the same age, weighing 18–22 g, were used in the study. The mice were purchased from Beijing Vitonglihua Laboratory Animal Center (purchase licence no. SCXK (Jing) 2021-0006, breeding licence no. SYXK (new) 2022-0003). All mice were housed under suitable environmental conditions, including a temperature of 18–25 °C, humidity of 50–60%, a 12-h light/dark cycle, and ad libitum access to food and water for one week.

### Construction of the HFD-induced AS Model

After one week of adaptation, 12 C57BL/6J mice were fed a normal diet to establish a control group, while the remaining ApoE^−/−^ mice (*n* = 60) were fed a high-fat diet (HFD) for 12 weeks. Subsequently, the mice were randomly divided into 5 groups (12 mice per group): model group (equal amount of distilled water, i.g), positive control group (3 mg·kg^−1^, i.g), JPHYP-H (56 g·kg^−1^, i.g), JPHYP-M (28 g·kg^−1^, i.g), and JPHYP-L (14 g·kg^−1^, i.g). The control group was fed a normal diet for 12 weeks, while the other groups were fed a HFD throughout the study period.

At the end of the experiment, the mice were anaesthetized with 10% chloral hydrate (400 mg/kg) via intraperitoneal injection. Blood was collected from the eyeballs of the mice and allowed to clot at room temperature for 2 h. The blood samples were then centrifuged at 4 °C and 3000 r·min^−1^ for 15 min, after which the resulting serum was collected and stored at −80 °C for further use. The entire aorta was removed, washed with saline, wrapped in tin foil, and frozen in liquid nitrogen for subsequent biomolecular experiments.

### Examination of Blood Lipids

The total cholesterol (TC), triglyceride (TG), low-density lipoprotein cholesterol (LDL-C), and high-density lipoprotein cholesterol (HDL-C) levels in the serum of mice from each group were determined using a fully automated biochemical instrument (Rayto Life and Analytical Sciences Co., Ltd., Model Chemray 240).

### Enzyme-linked Immunosorbent Assay

According to the instructions of the ELISA kit, detect TNF-α, IL-1β, IL-6 and MCP-1 in mouse serum (catalog numbers E-EL-M3063, E-EL-M0037c, E-EL-M0044c, E-EL-M3001) expression of inflammatory factors. In addition, ELISA was used to measure the levels of LPS (catalog number MM-0634M1) in mouse serum and cecal contents.

### Analysis of Atherosclerotic Lesions

Put the aorta into 4% polymerization fixing solution (catalog number 70100900, Biosharp, China) after 24–48 h, remove the aorta, put it in the oil red O working fluid at 45 °C Take it out and divide it in the 60% isopropanol solution until the outer wall of the outer vascular except the plaque becomes normal milky white. Take out the aorta tissue that is stained, carefully cut the aorta along the lumen, and fix it in the rubber On the board, the camera takes pictures. The root of the aorta is incubated in a 4% polymerized formaldehyde solution, frozen in the liquid nitrogen, cut into 5 mm slices, and performs HE, Masson and frozen slices of oil red O to stain. Essence use Image Pro Plus 6.0 to analyze the lesion area and size.

### Observation of Large Intestine Tissue Morphology

After the mouse experiment is completed and anesthetized, its abdomen is quickly incised, and a 3 cm portion of the terminal ileum located 5 cm behind the ileocecal area is taken out, washed with physiological saline, and then fixed with 4% formaldehyde, and then tissue The water was removed, then fixed with paraffin, cut, and stained with HE, Alcian blue and WGA.

### Immunohistochemical Analysis

The frozen sections were treated according to the instructions of the Servicebio Hypersensitive Two-step Kit. Primary antibodies (against TLR4, MyD88, JNK, p-JNK, AP-1, P38MAPK and p-P38MAPK) were added and incubated overnight at 4 °C. After rinsing with TBS, glyceraldehyde-3-phosphate dehydrogenase (GAPDH) was added to label the secondary antibody, and the samples were incubated at 37 °C for 20 min. DAB staining was performed, and the positive cells appeared brown-yellow. For optical microscopic observation, five fields of view were randomly selected from each section, and the positive cells were counted, with the mean value taken as the protein expression level expressed by the positive cell index (number of positive cells/total number of cells).

### Western Blotting Analysis

Lyse cultured macrophages, mouse aorta tissue and colon tissue according to the total protein extraction kit. At 100 °C, the protein is completely denatured, and the sample is diluted to 1× with 5× loading buffer and boiled for 10 min. Next, the lysates were subjected to SDS-PAGE gel electrophoresis and transferred to a 0.45 µm PVDF membrane. Block with 5% skim milk for 18 min at room temperature. Incubate primary antibodies (GAPDH (CST, 1:4000), β-actin (Servicebio, batch number AC220110015)) overnight at 4°C; TLR4 (Wuhan Proteintech, 66350-1-lg, 1:2000), MyD88 (Wuhan Proteintech, 67969-1-lg, 1:5000), JNK (Wuhan Proteintech, 66210-1-lg, 1:10000), p-JNK (Bioss, bsm-52462R, 1:1000), AP-1 (Wuhan Proteintech, 66590-1-lg, 1:20000), P38MAPK (Wuhan Proteintech, 1:2000), p-P38MAPK (Bioss, bs-5476R, 1:500), ZO-1 (Abcam, ab276131, 1:1000), Occludin1 (Wuhan Proteintech, 66378-1-Ig, 1:5000), Claudin1 (Abcam, ab307692, 1:1000), JAM-1 (Abcam, ab270446, 1:1000). Wash the membrane 6 times at room temperature, 5 min each time, add goat anti-mouse secondary antibody (Beijing Zhongshan Golden Bridge Biotechnology Co., Ltd., 224800105) and goat anti-rabbit secondary antibody (Wuhan Servicebio, CR2110082) for incubation, and use ultra-sensitive ECL chemistry After development with the luminescence kit (Wuhan Servicebio, CR2112141), the obtained protein bands were retained, and the results were analyzed using Image Pro Plus 6.0 software.

### Fecal Sample Collection and 16S rRNA Gene Sequencing

After the mice were dissected, the feces from the cecum were removed under sterile conditions. The total DNA of microbiome samples from different sources was extracted using the CTAB method. The quality of the DNA extraction was assessed using agar-gel electrophoresis, and DNA quantification was performed using a UV spectrophotometer. PCR amplification was carried out, and the purified PCR products were evaluated using an Agilent 2100 Bioanalyzer and Illumina’s library quantification kit. The qualified library concentration needed to be above 2 nM. The qualified online sequencing libraries (with nonrepetitive index sequences) were diluted in a gradient and mixed according to the required sequencing amount. The libraries were denatured into single strands by NaOH for on-machine sequencing. A NovaSeq 6000 sequencer was used for 2 × 250 bp double-ended sequencing with a NovaSeq 6000 SP Reagent Kit (500 cycles). The intestinal fecal genomes of the mice were sequenced for the V3-V4 variable region of the intestinal microbial 16S rRNA sequence by Shanghai BIOTRE Bioquant Biomedicine Technology Co., Ltd.

### Statistical Analysis

**Fig. 1:**
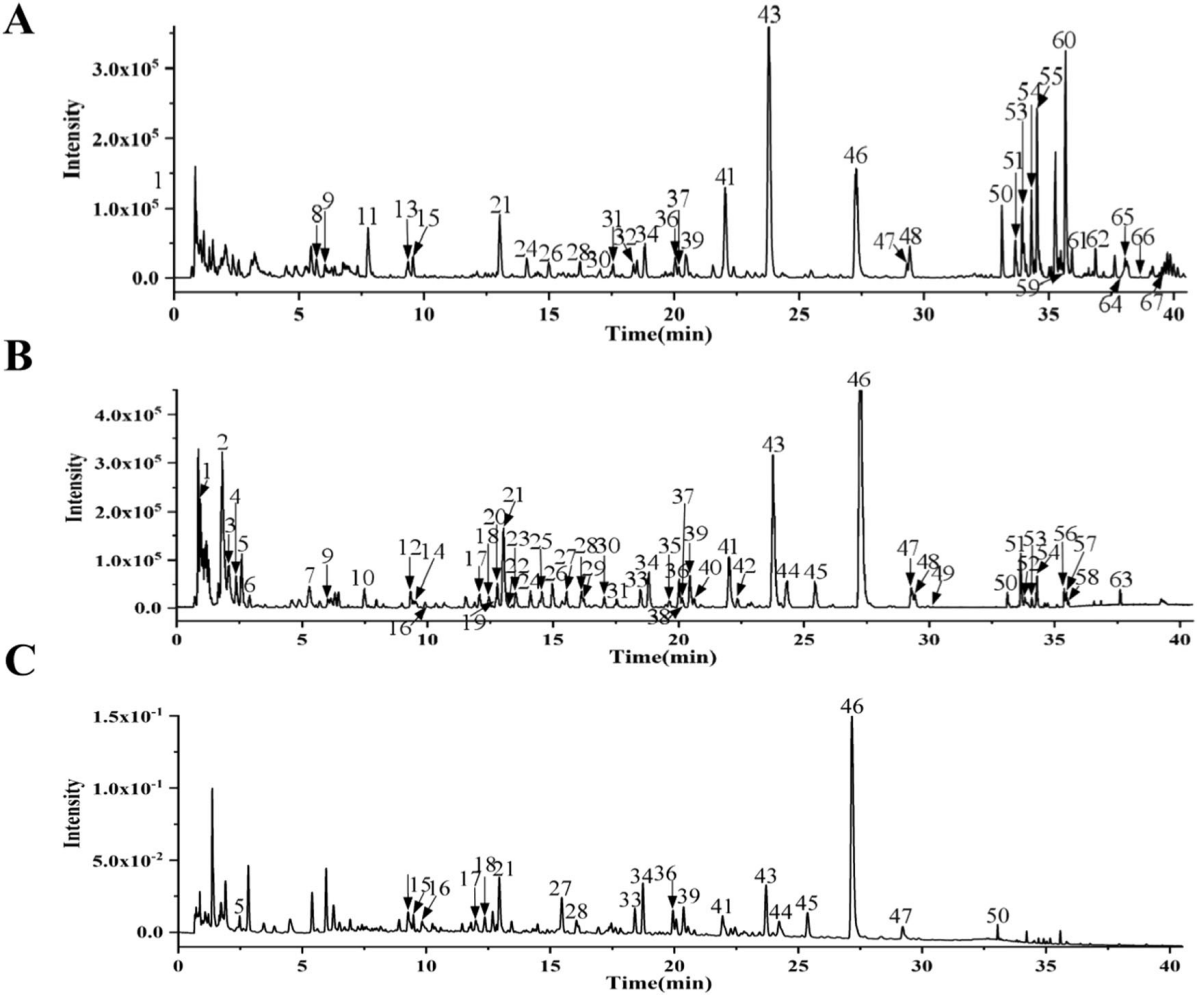
The chromatographic fingerprint of JPHYP. **A** Ion flow diagram of JPHYP in positive ion mode. **B** Ion flow diagram of JPHYP in negative ion mode. **C** UPLC UV chromatographic diagram of JPHYP with UV 254 nm. Molecular weight and formula of these compounds are listed in the Supplementary Material Table 3

The obtained experimental data indicates the average ± standard difference ($\bar{X}$ ± *S*) and *n* ≥ 3. Using SPSS Statistic 26.0 Software to analyze the significant differences between data, Origin 2021 and Graphpad Prism 9.5 Statistical Software Draw the pillar chart/folding diagram, independent samples of independent samples (Independent-Samples Test) analysis group differences (Two groups), one-way analysis of variance (one) analysis group differences (Three groups or more), when the result of *P* < 0.01 or *P* < 0.05 indicates that the difference is statistically significant Fig. 1.

## Results

### JPHYP Chemical Composition Analysis

UPLC-Q-TOF/MS was used to analyze JPHYP samples. Based on the multi-level mass spectrometry information of the samples, combined with the natural product high-resolution mass spectrometry database and related literature, 67 compounds were identified from the JPHYP samples (Table 3).

**Table 3:** Main components of Jianpi Huayu prescription sample identification results

No.	Time (min)	adduct ions	m/z	Molecular formula	English name	MS/MS data	CAS	Compound type
1	0.95	[M − H]^−^	191.0568	C_7_H_12_O_6_	Quinic acid	191.0567;173.0461;127.0402;93.0342;85.0292	77-95-2	Organic acids
2	1.82	[M − H]^−^	191.0205	C_6_H_8_O_7_	Citric acid	191.0193;129.0188;111.0083;87.0084;85.0291	77-92-9	Organic acids
3	2.07	[M − H]^−^	290.0884	C_11_H_17_NO_8_	N-Fructosyl pyroglutamate	200.0567;170.0456;128.0354	87251-82-9	Amino acids
4	2.37	[M + FA-H]^−^	711.2227	C_24_H_42_O_21_	Nystose	665.2176;485.1518;341.1092;323.0983;179.0563	13133-07-8	Carbohydrate
5	2.58	[M − H]^−^	499.1682	C_19_H_32_O_15_	/	499.1699;191.0566;173.0456;93.0350	/	/
6	2.90	[M − H]^−^	243.0629	C_9_H_12_N_2_O_6_	Uridine	243.0561;200.0569;152.0345;140.0341;128.0325;110.0241	58-96-8	Nucleosides
7	5.28	[M − H]^−^	827.2709	C30H52O26	Maltopentaose	827.2678;665.2188;503.1606;341.1088;179.0553;161.0433;101.0250	34620-76-3	Carbohydrate
8	5.48	[M + H]^+^	268.1050	C10H13N5O4	Adenosine	268.1017;136.0614;119.0344;94.0388;57.0337	58-61-7	Nucleosides
9	6.03	[M − H]^−^	282.0848	C_10_H_13_N_5_O_5_	Guanosine	282.0851;150.0425;133.0164;108.0215	118-00-3	Nucleosides
10	7.48	[M − H]^−^	197.0462	C_9_H_10_O_5_	Danshensu	197.0451;179.0348;135.0450;134.0368;123.0448	76822-21-4	Phenolic acids
11	7.76	[M + H]^+^	268.1547	C_14_H_21_NO_4_	Codonopsine	268.1545;161.0591;121.0636;88.0749	26989-20-8	Alkaloids
12	9.32	[M − H]^−^	611.1638	C_27_H_32_O_16_	Hydroxysafflor yellow B	611.1659;491.1221;473.1124;353.0545;328.0588;283.0620	78281-02-4	Flavonoid glycosides
13	9.36	[M + H]^+^	205.0978	C_11_H_12_N_2_O_2_	L-Tryptophan	188.0704;170.0602;146.0602;143.0728;118.0651;115.0540;91.0540	73-22-3	Amino acids
14	9.51	[M − H]^−^	137.0248	C_7_H_6_O_3_	Protocatechuic aldehyde	137.0239;119.0148;108.0217;92.0264;81.0338	139-85-5	Phenols
15	9.55	[M + H]^+^	251.1393	C_13_H_18_N_2_O_3_	Caffeoylputrescine	251.1387;234.1128;163.0392;145.0283;135.0440;117.0331;59.0385	29554-26-5	Alkaloids
16	9.98	[M − H]^−^	353.0876	C_16_H_18_O_9_	Neochlorogenic acid	353.0885;191.0560;179.0353;135.0448	906-33-2	Phenolic acids
17	12.06	[M − H]^−^	385.0793	C_16_H_18_O_11_	2-(E)-O-Feruloylgalactaric acid	385.0779;209.0298;191.0198;147.0295;85.0292	108043-98-7	Phenolic acids
18	12.44	[M − H]^−^	353.0893	C_16_H_18_O_9_	Chlorogenic acid	191.0565;85.0291	327-97-9	Phenolic acids
19	12.74	[M + FA-H]^−^	417.1419	C_17_H_24_O_9_	Syringin	417.1413;209.0812;194.0614;176.0462;161.0236	118-34-3	Phenolic glycosides
20	12.78	[M − H]^−^	239.0572	C_11_H_12_O_6_	Eucomic acid	239.0534;179.0.93;177.0707;1490630;133.0657;107.0490	60449-48-1	Phenolic acids
21	13.02	[M − H]^−^	611.1628	C_27_H_32_O_16_	Hydroxysafflor yellow A	611.1667;491.1231;173.1115;403.1055;325.0728;283.0620	78281-02-4	Flavonoid glycosides
22	13.12	[M − H]^−^	353.0886	C_16_H_18_O_9_	Cryptochlorogenic acid	353.0875;191.0553;179.0350;173.0451;135.0448;93.0339	905-99-7	Phenolic acids
23	13.52	[M − H]^−^	385.0785	C_16_H_18_O_11_	2-(E)-O-Feruloylglucaric acid	385.0780;209.0300;191.0195;147.0293;129.0190;85.0295	121210-29-5	Phenolic acids
24	14.10	[M − H]^−^	577.1370	C_30_H_26_O_12_	Procyanidin B1	577.1358;451.1046;425.0879;407.0778;339.0858;289.0710;245.0805;161.0239;125.0237	20315-25-7	Tannins
25	14.56	[M + FA-H]^−^	431.1942	C_19_H_30_O_8_	Roseoside	431.1948;385.1894;223.1355;205.1235;161.0454;153.0928	54835-70-0	Monoterpene glycosides
26	14.98	[M − H]^−^	289.0724	C_15_H_14_O_6_	Catechin	289.0724;245.0828;203.0714;151.0419;125.0246;123.0444;109.0294	490-46-0	Flavonoids
27	15.54	[2M − H]^−^	591.1011	C_13_H_12_O_8_	Caffeoylmalic Acid	295.0465;179.0356;133.0149;115.0042	149197-97-7	Phenolic acids
28	16.13	[M − H]^−^	325.0939	C_15_H_18_O_8_	p-Coumaroylglucose	163.0386;119.0497	7139-64-2	Phenolic glycosides
29	16.22	[M − H]^−^	593.1543	C_27_H_30_O_15_	Vicenin-2	593.1563;503.1226;473.1126;395.0791;383.0796;353.0677	23666-13-9	Flavonoid glycosides
30	17.04	[M − H]^−^	623.1632	C_28_H_32_O_16_	Diosmetin-6,8-di-C-glucoside	623.1667;533.1313;503.1205;413.0865;383.0774	98813-28-6	Flavonoid glycosides
31	17.54	[M − H]^−^	625.1438	C_27_H_30_O_17_	6-Hydroxykaempferol 3,6-diglucoside	625.1433;463.0922;300.0274;271.0250	142674-16-6	Flavonoid glycosides
32	18.38	M^+^	350.1970	C_19_H_28_NO_5_^+^	Codonopyrrolidium A	350.1914;250.1422;205.0861;161.0588;88.0746	1122492-83-4	Alkaloids
33	18.48	[M − H]^−^	609.1487	C_27_H_30_O_16_	Rutin	609.1527;300.0294;271.0270;255.0312	153-18-4	Flavonoid glycosides
34	18.81	[M − H]^−^	609.1485	C_27_H_30_O_16_	Quercetin 3-O-rhamnoside 7-O-glucoside	609.1510;300.0285;271.0262;255.0299	17306-45-5	Flavonoid glycosides
35	19.64	[M − H]^−^	649.2534	C_32_H_42_O_14_	Limonin glycoside	649.2529;605.2648;443.2080;347.1854;209.0820	1180-71-8	Limonoids
36	20.02	[M − H]^−^	593.1536	C_27_H_30_O_15_	Kaempferol 3-O-robinobioside	593.1523;284.0322;255.0289;227.0350	17297-56-2	Flavonoid glycosides
37	20.15	[M − H]^−^	609.1491	C_27_H_30_O_16_	Quercetin-3-O-neohesperidoside	609.1487;300.0277;271.0247;255.0299	32453-36-4	Flavonoid glycosides
38	20.17	[M − H]^−^	463.0895	C_21_H_20_O_12_	Hyperoside	463.0922;300.0277;271.0248;255.0291;143.0313	482-36-0	Flavonoid glycosides
39	20.45	[M − H]^−^	593.1515	C_27_H_30_O_15_	Kaempferol-3-O-rutinoside	593.1567;284.0341255.0313;227.0357	17650-84-9	Flavonoid glycosides
40	20.62	[M − H]^−^	463.0897	C_21_H_20_O_12_	Isoquercitrin	463.0893;300.0272;271.0251;255.0292;243.0297	482-35-9	Flavonoid glycosides
41	22.02	[M − H]^−^	579.1740	C_27_H_32_O_14_	Naringin	579.1740;313.0709;271.0605;151.0036;119.0482	14259-46-2	Flavonoid glycosides
42	22.35	[M − H]^−^	593.1537	C_27_H_30_O_15_	Kaempferol-3-O-neohespeidoside	593.1570;285.0414;255.0315	32602-81-6	Flavonoid glycosides
43	23.78	[M − H]^−^	609.1848	C_28_H_34_O_15_	Hesperidin	609.1883;301.0725;286.0487	520-26-3	Flavonoid glycosides
44	24.31	[M − H]^−^	359.0783	C_18_H_16_O_8_	Rosmarinic acid	359.0802;197.0462;179.0356;161.0248;133.0297	20283-92-5	Phenolic acids
45	25.45	[M − H]^−^	537.1058	C_27_H_22_O_12_	Lithospermic acid	493.1181;313.0715;295.0620;185.0246;109.0292	28831-65-4	Phenolic acids
46	27.25	[M − H]^−^	717.1502	C_36_H_30_O_16_	Salvianolic acid B	717.1537;519.0995;339.0548;321.0466;295.0631	121521-90-2	Phenolic acids
47	29.30	[M − H]^−^	717.1502	C_36_H_30_O_16_	Salvianolic acid Y	717.1519;519.0973;339.0522;321.0414;295.0624	1638738-76-7	Phenolic acids
48	29.42	[M − H]^−^	593.1915	C_28_H_34_O_14_	Poncirin	593.1922;309.0779;285.0783;164.0110	14941-08-3	Flavonoid glycosides
49	30.81	[M + FA-H]^−^	723.3644	C36H54O12	Arvenin III	723.3676;677.3586;659.3474;641.3396;593.3004;575.2927	65597-45-7	Triterpene saponins
50	33.13	[M + H]^+^	725.2301	C_33_H_40_O_18_	Natsudaidain-3-O-[3-hydroxy-3-methylglutarate(1 → 6)]-β-glucoside.	725.2305;419.1340;404.1109;389.0875	1179358-72-5	Flavonoid glycosides
51	33.65	[M + H]+	1095.5963	C_53_H_90_O_23_	Gypenoside LVI	1095.5956;915.5333;783.4907621.4365;459.3844;441.3735	105214-48-0	Triterpene saponins
52	33.81	[M − H]^−^	329.2344	C_18_H_34_O_5_	9,12,13-Trihydroxy-10-octadecanoic acid	329.2346;229.1454;211.1349;171.1032	29907-56-0	Fatty acids
53	33.93	[M + FA-H]^−^	1007.5451	C_48_H_82_O_19_	Gypenoside XLVI	1007.5527;961.5473;799.4928;637.4376;475.3820;221.0675;161.0453	94705-70-1	Triterpene saponins
54	34.29	[M + FA-H]^−^	549.3450	C_30_H_48_O_6_	16-oxoalisol A	549.3479;503.3422;485.3313;455.2829;427.2886;367.2660	124515-98-6	Triterpenoids
55	35.26	[M + H]^+^	403.1389	C_21_H_22_O_8_	Nobiletin	403.1404;388.1166;373.0930;358.0697;327.0873;301.0727;211.0248;183.0292	478-01-3	Flavonoids
56	35.38	[M + FA-H]^−^	845.4960	C_42_H_72_O_14_	Gypenoside L	845.4996;799.4927;637.4383;475.3832	94987-09-4	Triterpene saponins
57	35.47	[M + FA-H]^-^	845.4960	C_42_H_72_O_14_	Gypenoside LI	845.5004;799.4950;637.4399;475.3834	94987-10-7	Triterpene saponins
58	35.51	[M + FA-H]-	603.3202	C_32_H_46_O_8_	Cucurbitacin B	603.3215;557.3108;539.3259;497.2953;481.3060;411.2230;385.2032;301.1416;273.1509	6199-67-3	Triterpenoids
59	35.65	[M + H]^+^	249.1494	C_15_H_20_O_3_	AtractylenolideIII	/	73030-71-4	Sesquiterpenes
60	35.66	[M + H]^+^	433.1496	C_22_H_24_O_9_	3’,4’,3,5,6,7,8-Heptamethoxyflavone	433.1496;418.1256;403.1027;385.0924;373.0536	1178-24-1	Flavonoids
61	35.92	[M + H]^+^	373.1297	C_20_H_20_O_7_	Tangeretin	373.1292;358.1055;343.0822;325.0706;315.0877;300.0630	481-53-8	Flavonoids
62	36.87	[M + H]^+^	529.3540	C_32_H_48_O_6_	23-Acetyl alisol C	529.3537;511.3437;469.3328;451.3223;415.2853	26575-93-9	Triterpenoids
63	37.61	[M + FA-H]^-^	535.3672	C_30_H_50_O_5_	Alisol A	535.3671;489.3607;471.3498;339.2685	19885-10-0	Triterpenoids
64	38.03	[M + H]^+^	277.0861	C_18_H_12_O_3_	Tanshinone I	/	568-73-0	Diterpene quinones
65	38.05	[M + H]^+^	297.1489	C_19_H_20_O_3_	Cryptotanshinone	297.1491;179.1381;268.1088;254.0939;251.1431	35825-57-1	Diterpene quinones
66	38.66	[M + H]^+^	515.3720	C_32_H_50_O_5_	23-Acetyl alisol B	515.3746;457.3338;419.3257;383.2956;339.2663;219.1758	561055-03-6	Triterpenoids
67	39.58	[M + H]^+^	295.1330	C_19_H_18_O_3_	Tanshinone IIA	295.1343;277.1231;262.0992;249.1287;191.0858	568-72-9	Diterpene quinones

### Effect of JPHYP on Macrophage Cell Viability

**Fig. 2:**
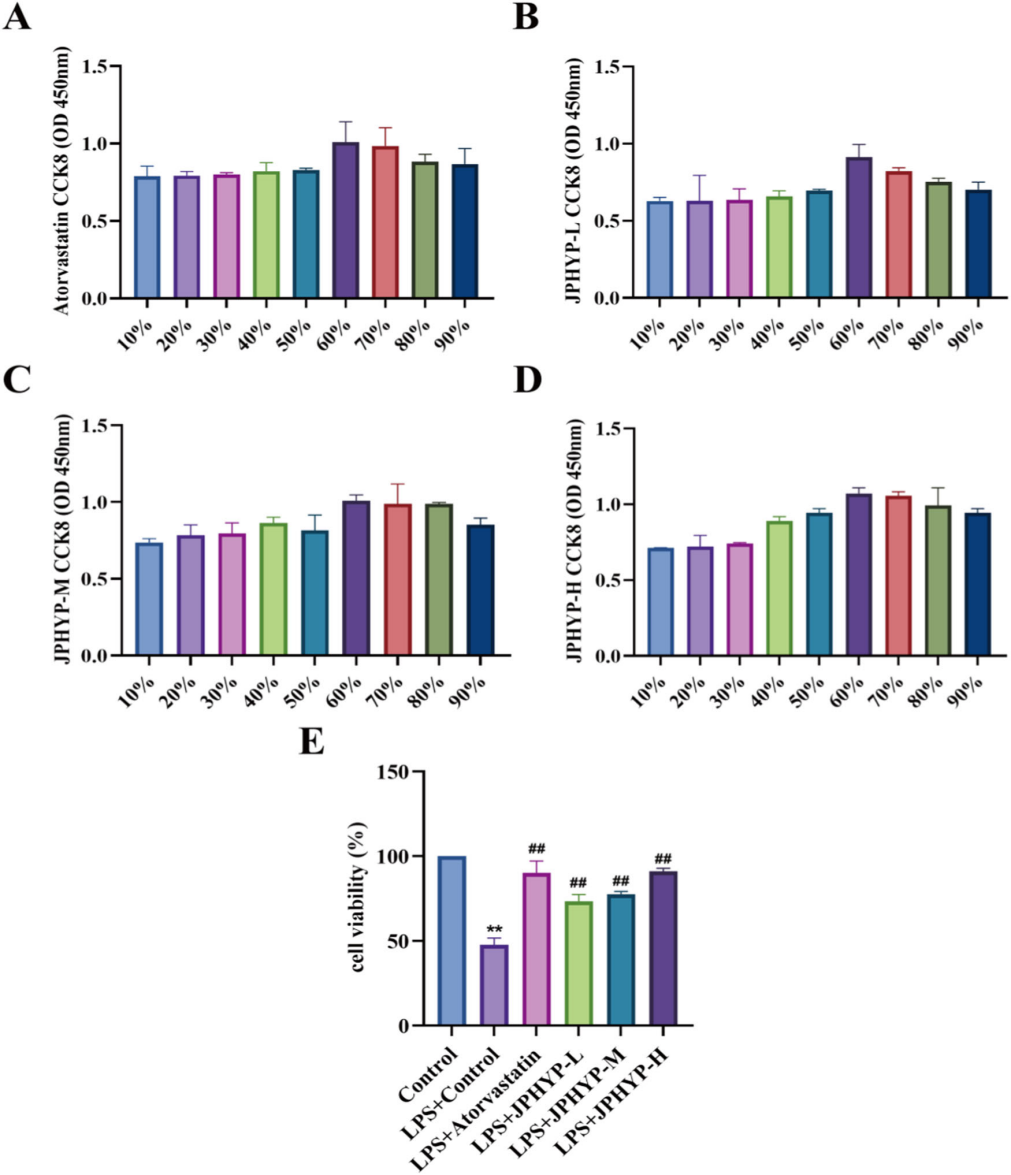
Effect of JPHYP on LPS-induced cell viability of RAW264.7 macrophages. **A**–**D** CCK-8 determines the optimal dilution ratio of drug-containing serum. **E** Effect of JPHYP medicated serum on macrophage viability (*n* = 3). Data are expressed as mean ± SD (standard deviation). *p < 0.05, **p < 0.01 vs Control; ^#^*p* < 0.05, ^##^*p* < 0.01 vs Model

To ascertain the proper drug dosage concentration, the impact of JPHYP on the viability of LPS-induced RAW264.7 macrophages was assessed using CCK-8. In the ensuing trials, the medicated serum of the atorvastatin, JPHYP-L, JPHYP-M, and JPHYP-H groups was diluted by 60% because it was evident that this resulted in the best cell viability (Fig. 2A–D).

The amount of LPS-induced cell damage was lessened in RAW264.7 cells when they were pretreated with JPHYP serum, and this reduction was dose-dependent. In essence, the JPHYP-H group’s serum cell activity leveled back down to that of the control group. The JPHYP-L and JPHYP-M groups’ serums both strongly prevented the LPS-induced decline in cell activity (Fig. 2E).

### JPHYP Reduces ROS Generation in Macrophages

**Fig. 3:**
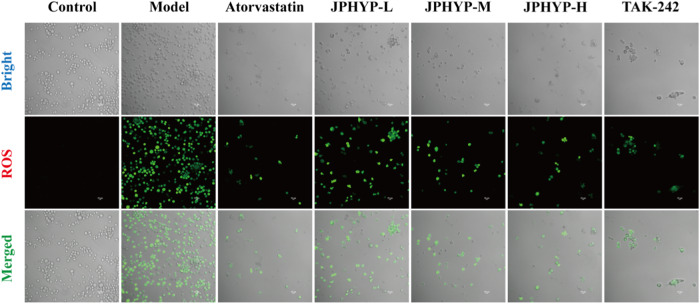
JPHYP reduces macrophage ROS levels (40×)

GLSM was utilized to identify variations in JPHYP-produced ROS levels in LPS-induced RAW264.7 macrophages. When LPS was applied to RAW264.7 macrophages, the intracellular ROS fluorescence intensity in the model group was much higher than in the control group, suggesting that LPS caused the macrophages to create a significant amount of reactive oxygen species. The intracellular ROS levels were significantly reduced following a 24-hour pretreatment with low, medium, and high doses of JPHYP and TLR4 blocker. This suggests that JPHYP can reduce LPS-induced reactive oxygen species in RAW264.7 macrophages, and that the ROS level decreases more obviously as the dose of JPHYP increases (Fig. 3).

### JPHYP has Lipid-lowering and Anti-inflammatory Effects

**Fig. 4:**
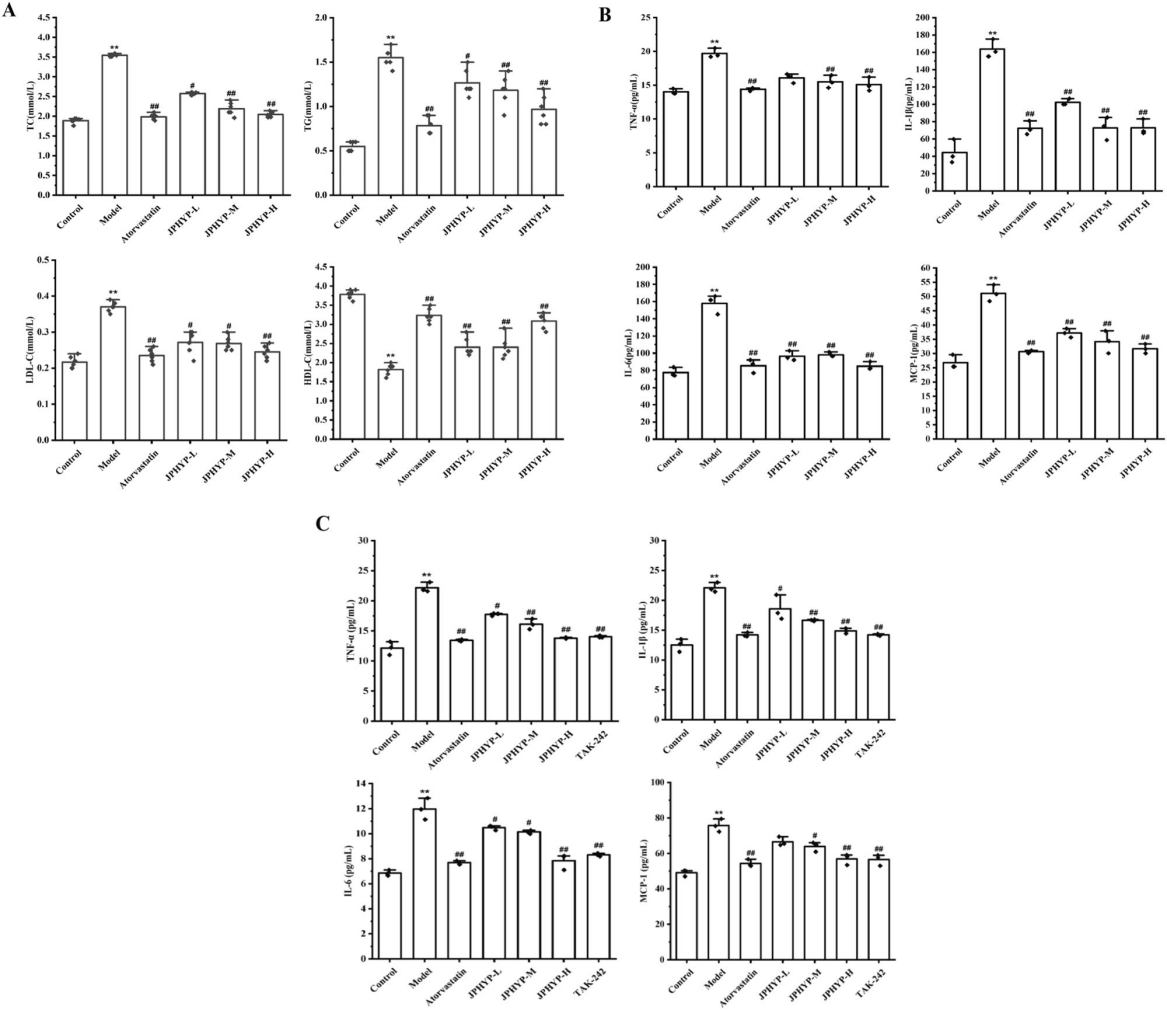
JPHYP improves blood lipids and inflammation levels. **A** All natural biochemical instrument of TG, TC, LDL-C and HDL-C in mice serum (mmol/L) (*n* = 6). **B** ELISA of TNF-α, IL-1β, IL-6 and MCP-1 (pg/ml) in ApoE^−/−^ mice serum (*n* = 3). **C** ELISA of TNF-α, IL-1β, IL-6 and MCP-1 (pg/mL) in macrophages (*n* = 3). Data are expressed as mean ± SD (standard deviation). *p < 0.05, **p < 0.01 vs Control; ^#^*p* < 0.05, ^##^*p* < 0.01 vs Model

Compared with the control group, the TC, TG and LDL-C levels of mice in the model group increased, and the serum HDL-C levels decreased (Fig. 4A). The levels of inflammatory factors TNF-α, IL-1β, IL-6 and MCP-1 in cells and serum increased. JPHYP intervention reversed these changes to varying degrees. In particular, the JPHYP-H group had a significant improvement effect, which was similar to atorvastatin treatment (Fig. 4B, C).

### JPHYP Reduces LPS Production in ApoE^−/−^ Mice

**Fig. 5:**
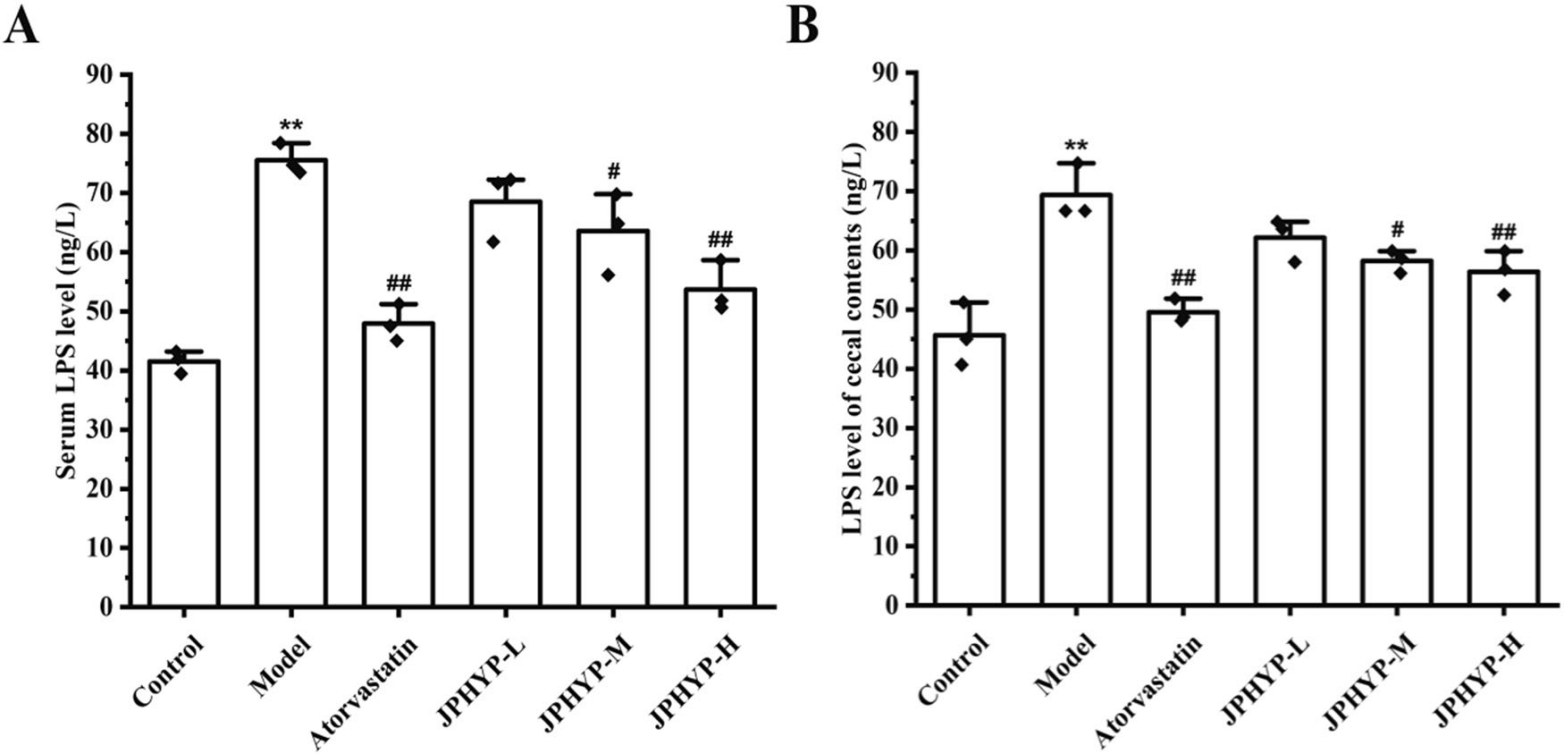
LPS levels in serum and cecum contents of mice in each group (*n* = 3). **A** LPS content in serum of ApoE^−/−^ mice. **B** LPS content in cecal contents of ApoE^−/−^ mice. Data are expressed as mean ± SD (standard deviation). *p < 0.05, **p < 0.01 vs Control; ^#^*p* < 0.05, ^##^*p* < 0.01 vs Model

The amount of LPS in mouse serum and cecal fluids was measured using ELISA. The findings demonstrated that while the levels of LPS in the serum and cecal contents of mice in the JPHYP medium-dose and high-dose groups were much lower than those in the model group, the levels of LPS in the animals in the model group were significantly higher (Fig. 5A, B).

### JPHYP Reduces Atherosclerotic Lesions in ApoE^−/−^ Mice

**Fig. 6:**
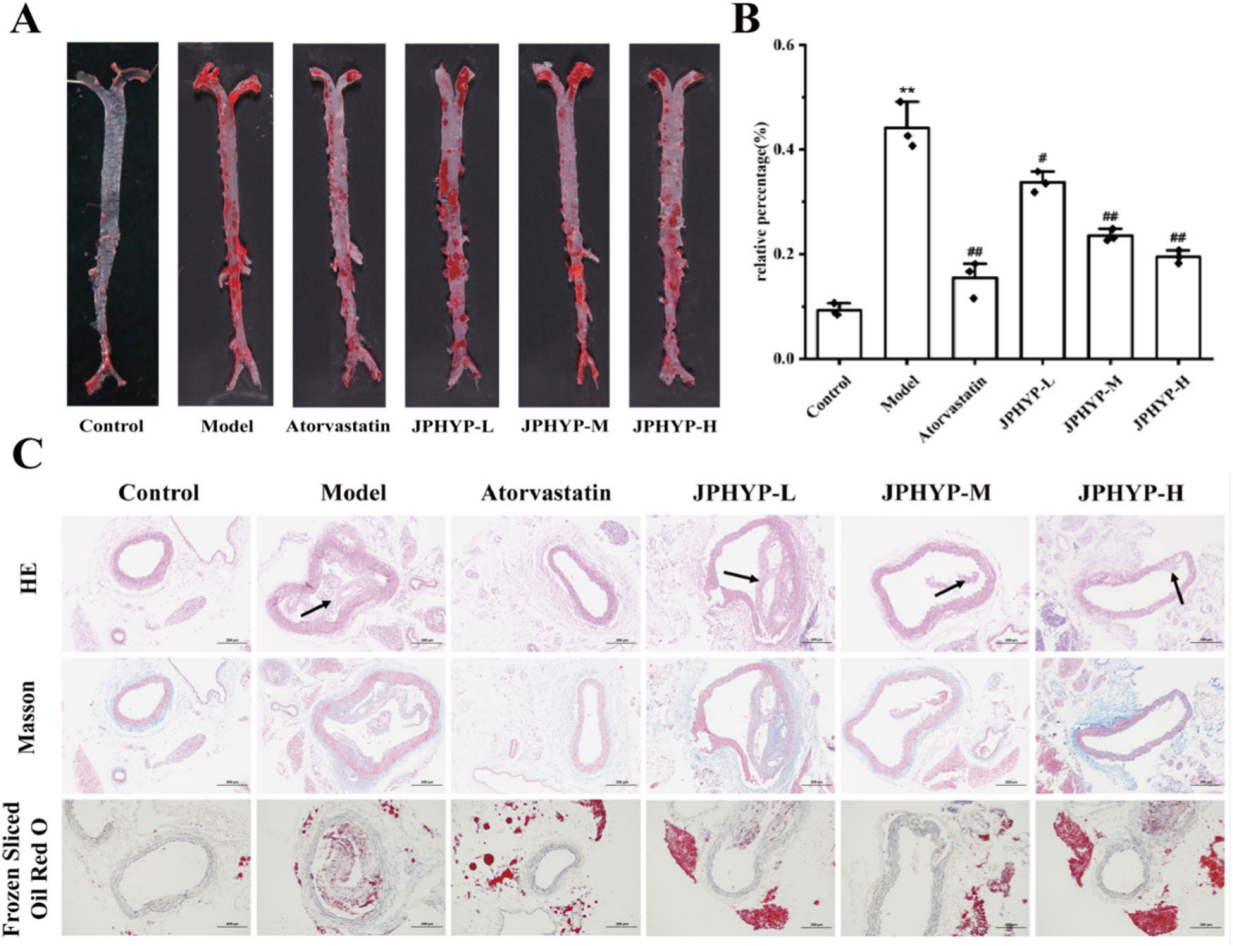
Ameliorative effect of JPHYP on atherosclerosis in ApoE^−/−^ mice. **A**, **B** Representative photographs of the whole aorta by Oil Red O staining and quantitative analysis of plaque area of the whole aorta (*n* = 3). **C** Representative images of aortic root sections by HE staining. Representative images of aortic root sections by Masson staining. Representative images of aortic root sections by frozen slices Oil Red O staining. Data are expressed as mean ± SD (standard deviation). *p < 0.05, **p < 0.01 vs Control; ^#^*p* < 0.05, ^##^*p* < 0.01 vs Model

In the experiment mentioned above, Oil Red O was used to stain the whole aorta of the ApoE^−/−^ mice. In the model group, the inner wall of the aorta was significantly thicker and there were numerous arterial plaques distributed at the renal artery bifurcation and aortic arch. The longitudinal section of the aorta in the control group revealed that there was no obvious plaque on the inner wall of the blood vessel (9.28%), and the inner wall was smooth and uniform. The aortic arch in the atorvastatin group (15.45%) and the JPHYP administration group (JPHYP-L: 33.73%, JPHYP-M: 23.51%, JPHYP-H: 19.46%) had plaques, although the area of the plaques was much smaller than in the model group. The block area increased significantly (44.17%) (Fig. 6A, B).

Aortic sinus staining revealed that the mice in the model group had considerably worsened atherosclerosis lesions, and HE, Masson, and Oil Red O staining of frozen sections revealed atherosclerotic lesions in the aortic root section. The mice in the JPHYP-L and JPHYP-M groups had significantly less atherosclerotic lesions than the mice in the model group; however, the effect was not as significant as it was in the JPHYP-H group. This suggests that JPHYP’s intervention effect increased in a dose-dependent way (Fig. 6C). According to the data above, JPHYP therapy decreased atherosclerotic lesions brought on by a high fat diet.

### JPHYP Improves Changes in Large Intestinal Tissue Morphology and Mucin in ApoE^−/−^ Mice

The results of HE staining demonstrated that the mice in the control group had intact large intestinal tissue structure, uniformly distributed intestinal muscularis propria, and a high number of goblet cells in the mucosal layer; in contrast, the mice in the model group had damaged large intestinal tissue, thinner intestinal muscularis propria, and a mucosal layer with many goblet cells. Goblet cell count dropped; damage to large intestine tissue was markedly improved, intestinal muscularis propria was repaired, and goblet cell count in the mucosal layer rose following JPHYP administration intervention.

**Fig. 7:**
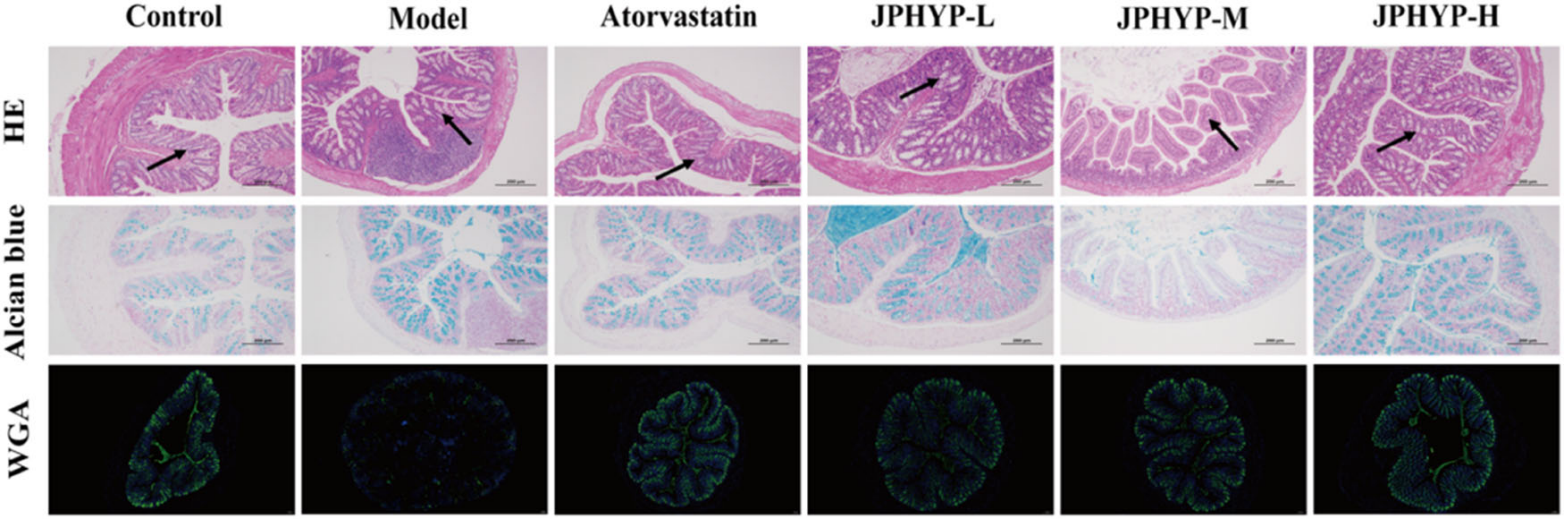
JPHYP improves large intestinal tissue morphology and mucin changes in ApoE^−/−^ mice (100×)

The intestinal barrier’s mucin was stained using alcian blue staining. The outcomes demonstrated that JPHYP had a substantial impact on the mucin secretion and goblet cell count in the mouse large intestine tissue, however both were much lower in the model group than in the control group. Mucin expression can be considerably restored by the protective effect. In addition, WGA was employed in this work to detect mucin in the intestinal barrier through tissue fluorescence. The green fluorescent dye FITC was employed to label WGA in order to stain and observe the mucin following its particular binding to the polysaccharide portion of the mucin. The findings demonstrated that the model group’s mice’s tissue fluorescence was significantly lower than that of the control group’s mice, suggesting that the expression of mucopolysaccharides was down-regulated. However, following intervention with the administration of JPHYP, the expression of mucopolysaccharides was significantly restored (Fig. 7).

### JPHYP Improves Intestinal Barrier Damage by Regulating the Expression of ZO-1, Occludin1, Claudin1 and JAM-1 Proteins in ApoE^−/−^ Mice

**Fig. 8:**
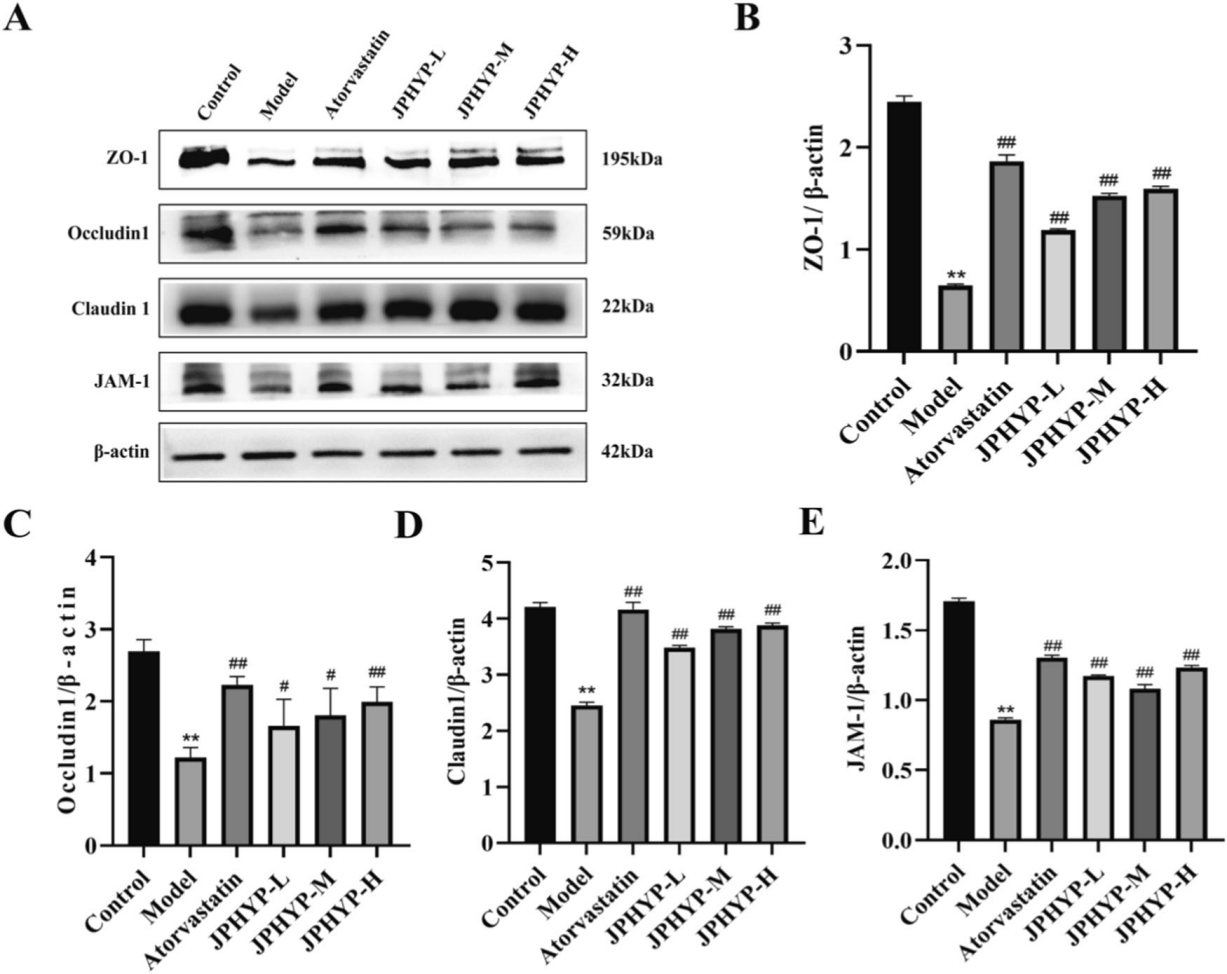
JPHYP improves intestinal barrier damage in ApoE^−/−^ mice through intestinal tight junction proteins. **A**–**E** Western blot of ZO-1, Occludin1, Claudin1 and JAM-1 in ApoE^−/−^ mice colon. Protein quantification is shown in bar charts (*n* = 3). Data are expressed as mean ± SD (standard deviation). *p < 0.05, **p < 0.01 vs Control; ^#^*p* < 0.05, ^##^*p* < 0.01 vs Model

Adjacent endothelial cells’ tight connections have the ability to alter vascular homeostasis. Furthermore, intercellular tight junction structures create an intestine permeability barrier that prevents harmful chemicals from entering the bloodstream and causing inflammation. JAM-1, Occludin1, ZO-1, Claudin1, and Claudin1 are examples of junction proteins that keep endothelial junctions permeable. ZO-1, Occludin1, Claudin1, and JAM-1 protein expression levels in the intestinal tissue of the JPHYP group were considerably higher than those of the high-fat diet group in the current study (Fig. 8A–E), suggesting that JPHYP can enhance enteral functioning in ApoE^−/−^ mice.

### JPHYP Improves Inflammation in Macrophages and ApoE^−/−^ Mice through TLR4/MyD88/MAPK Signaling Pathway

**Fig. 9:**
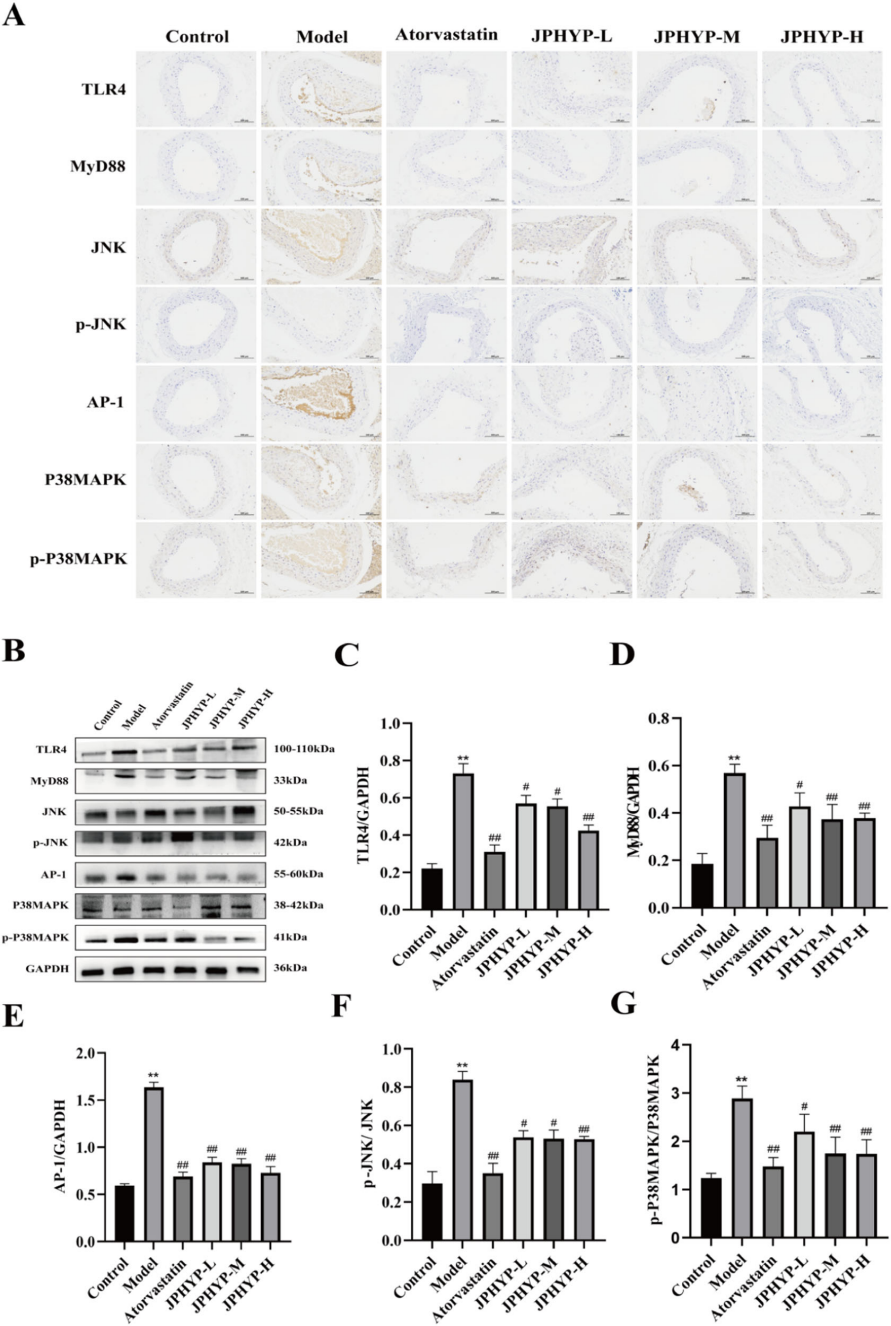
JPHYP improves inflammation in ApoE mice through TLR4/MyD88/MAPK signaling pathway. **A** The expression of TLR4/MyD88/MAPK signaling pathway in aorta of ApoE^−/−^ mice with atherosclerosis was detected by immunohistochemical method (200×). **B**–**G** Western blot of TLR4, MyD88, p-JNK, AP-1 and p-P38MAPK in ApoE^−/−^ mice aorta. Protein quantification is shown in bar charts (*n* = 3). Data are expressed as mean ± SD (standard deviation). *p < 0.05, **p < 0.01 vs Control; ^#^*p* < 0.05, ^##^*p* < 0.01 vs Model

To find out whether JPHYP reduces inflammation in macrophages and AS mice via the TLR4/MyD88/MAPK signaling pathway, immunohistochemistry and Western blot were employed. The positive expression of TLR4, MyD88, JNK, p-JNK, AP-1, P38MAPK, and p-P38MAPK was primarily observed as brown-yellow particles in the cytoplasm or membrane, and it was expressed inside the plaque, according to the results of in vivo immunohistochemistry. In the aorta of experimental animals, the positive reaction intensity of TLR4, MyD88, JNK, p-JNK, AP-1, P38MAPK, and p-P38MAPK is displayed (Table 4, Fig. 9A): blue denotes negative expression, brown shows positive expression. Positive expression was nearly nonexistent in the control group and highly prevalent in the model and treatment groups. The positive expression of TLR4, MyD88, JNK, p-JNK, AP-1, P38MAPK, and p-P38MAPK was equal in the atorvastatin group and the JPHYP-H group, while the positive expression of each treatment group was lower than that of the model group.

**Table 4:** Mouse TLR4, MyD88, JNK, p-JNK, AP-1, P38MAPK and p-P38MAPK in each group comparison of average optical density values ($\bar{X}$ ± *S, n* = 3)

Group	TLR4	MyD88	JNK	p-JNK	AP-1	P38MAPK	p-P38MAPK
Control	1.65 ± 0.07	1.44 ± 0.05	3.23 ± 0.14	1.94 ± 0.08	1.48 ± 0.04	1.75 ± 0.06	2.56 ± 0.11
Model	19.56 ± 0.50^**^	16.12 ± 0.61^**^	28.41 ± 0.72^**^	20.04 ± 0.66^**^	19.80 ± 0.52^**^	24.42 ± 0.83^**^	23.22 ± 0.54^**^
Atorvastatin	1.80 ± 0.06^##^	1.54 ± 0.06^##^	3.68 ± 0.17^##^	1.98 ± 0.08^##^	1.80 ± 0.07^##^	2.32 ± 0.09^##^	2.61 ± 0.13^##^
JPHYP-L	5.42 ± 0.26^##^	3.02 ± 0.13^##^	10.79 ± 0.48	4.01 ± 0.27^#^	3.24 ± 0.14^##^	5.57 ± 0.28^#^	8.65 ± 0.28^#^
JPHYP-M	2.22 ± 0.08^##^	1.84 ± 0.09^##^	4.13 ± 0.19^##^	3.20 ± 0.07^#^	2.66 ± 0.10^##^	3.37 ± 0.10^##^	5.02 ± 0.17^#^
JPHYP-H	1.82 ± 0.08^##^	1.61 ± 0.07^##^	3.86 ± 0.18^##^	2.55 ± 0.12^##^	1.86 ± 0.05^##^	2.38 ± 0.08^##^	3.07 ± 0.15^##^

^*^p < 0.05, **p < 0.01 vs Control; ^#^*p* < 0.05, ^##^*p* < 0.01 vs Model

**Fig. 10:**
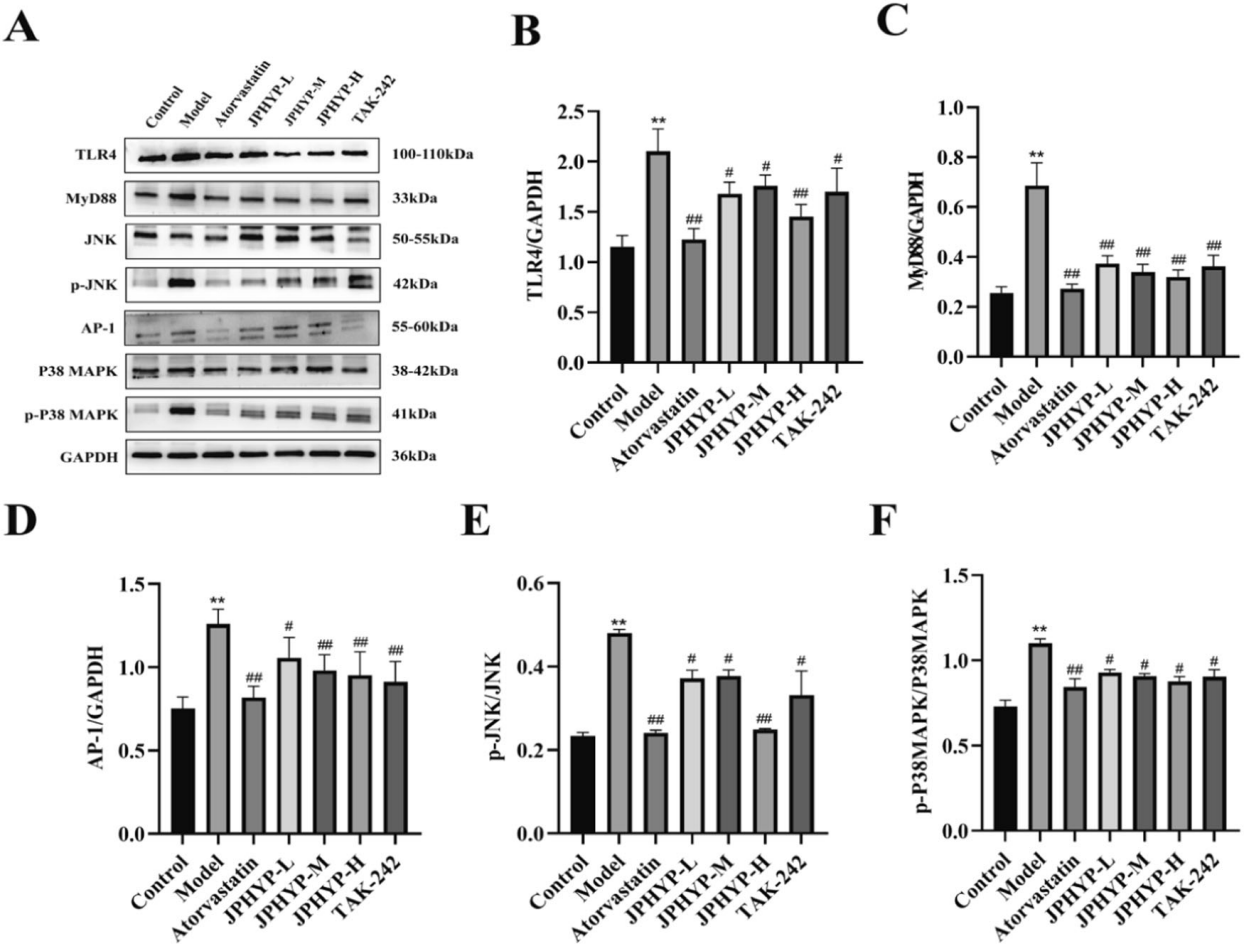
JPHYP improves macrophage inflammation through TLR4/MyD88/MAPK signaling pathway. **A**–**F** Western blot of TLR4, MyD88, p-JNK, AP-1 and p-P38MAPK in macrophage. Protein quantification is shown in bar charts (*n* = 3). Data are expressed as mean ± SD (standard deviation). *p < 0.05, **p < 0.01 vs Control; ^#^*p* < 0.05, ^##^*p* < 0.01 vs Model

The model group had significantly higher protein levels of TLR4, MyD88, JNK, p-JNK, AP-1, P38MAPK, and p-P38MAPK, according to in vitro and in vivo Western blot data; in contrast, JPHYP reduced the levels of TLR4, MyD88, JNK, p-JNK, AP-1, P38MAPK, and p-P38MAPK (Figs. 9B–G and 10A–F). These findings suggest that by blocking the TLR4/MyD88/MAPK signaling pathway, JPHYP may be used to prevent and treat AS.

### JPHYP Regulates Intestinal Microbiota Dysbiosis in ApoE^−/−^ Mice

**Fig. 11:**
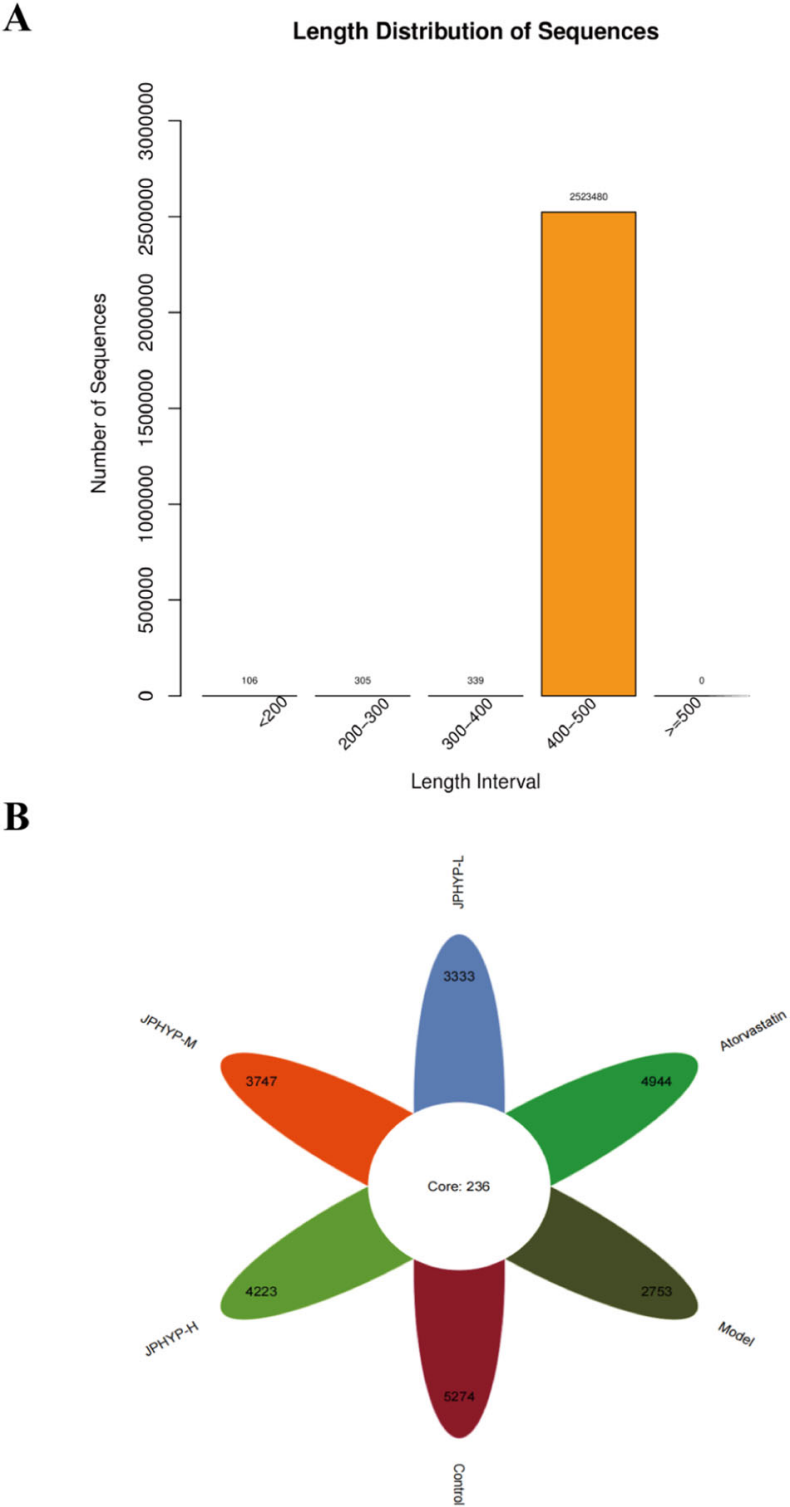
JPHYP changes the number of intestinal microbiota species in ApoE^−/−^ mice. **A** Clean reads based on 16S rRNA analysis. **B** Venn diagram between mouse intestinal flora species groups

**Fig. 12:**
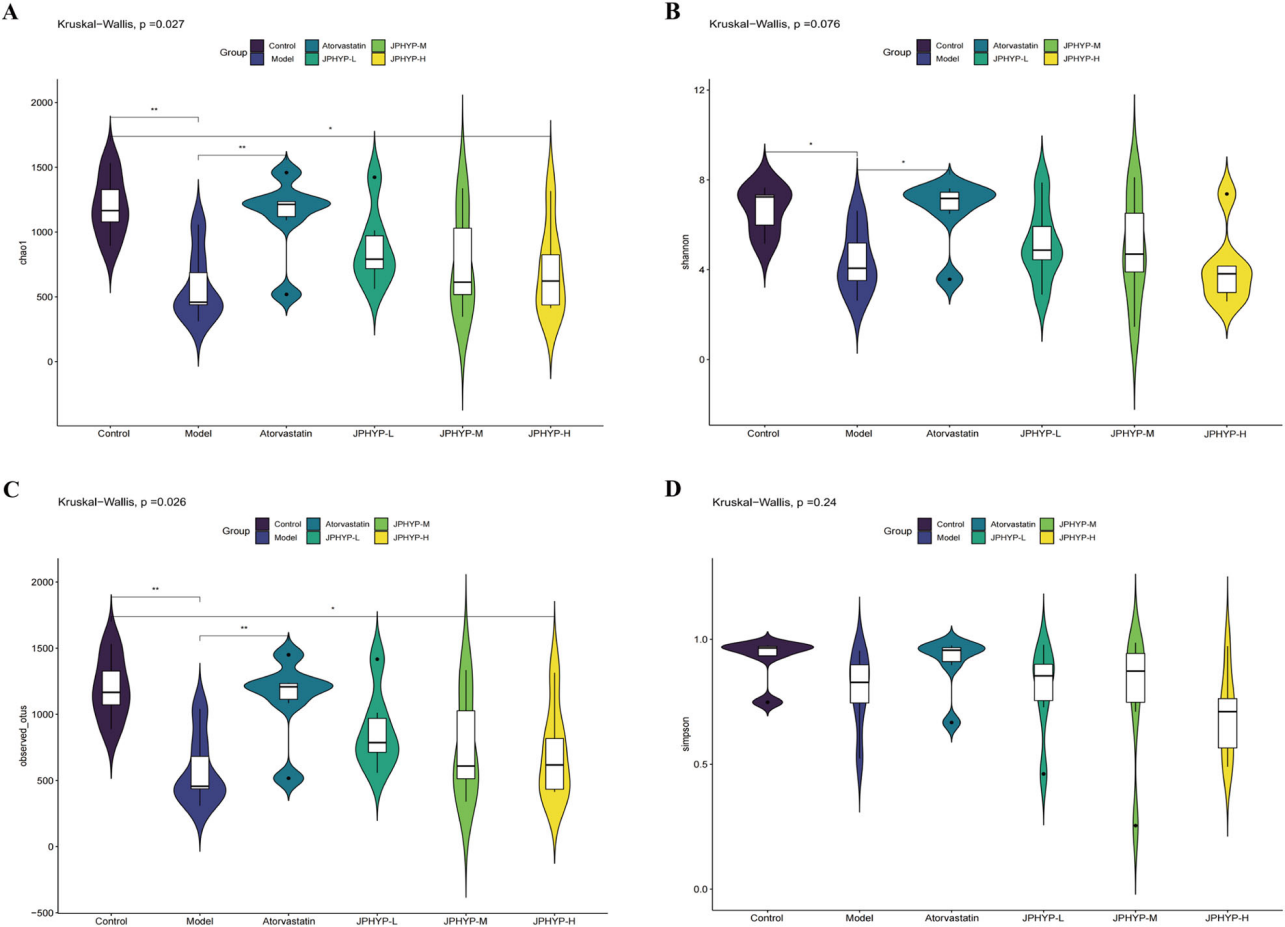
JPHYP restores alpha diversity of intestinal microbiota in AS mice (*n* = 6). **A** Chao1 index; **B** Shannon index; **C** Observed_otus index; **D** Simpson index. Data are expressed as mean ± SD (standard deviation). *p < 0.05, **p < 0.01

**Fig. 13:**
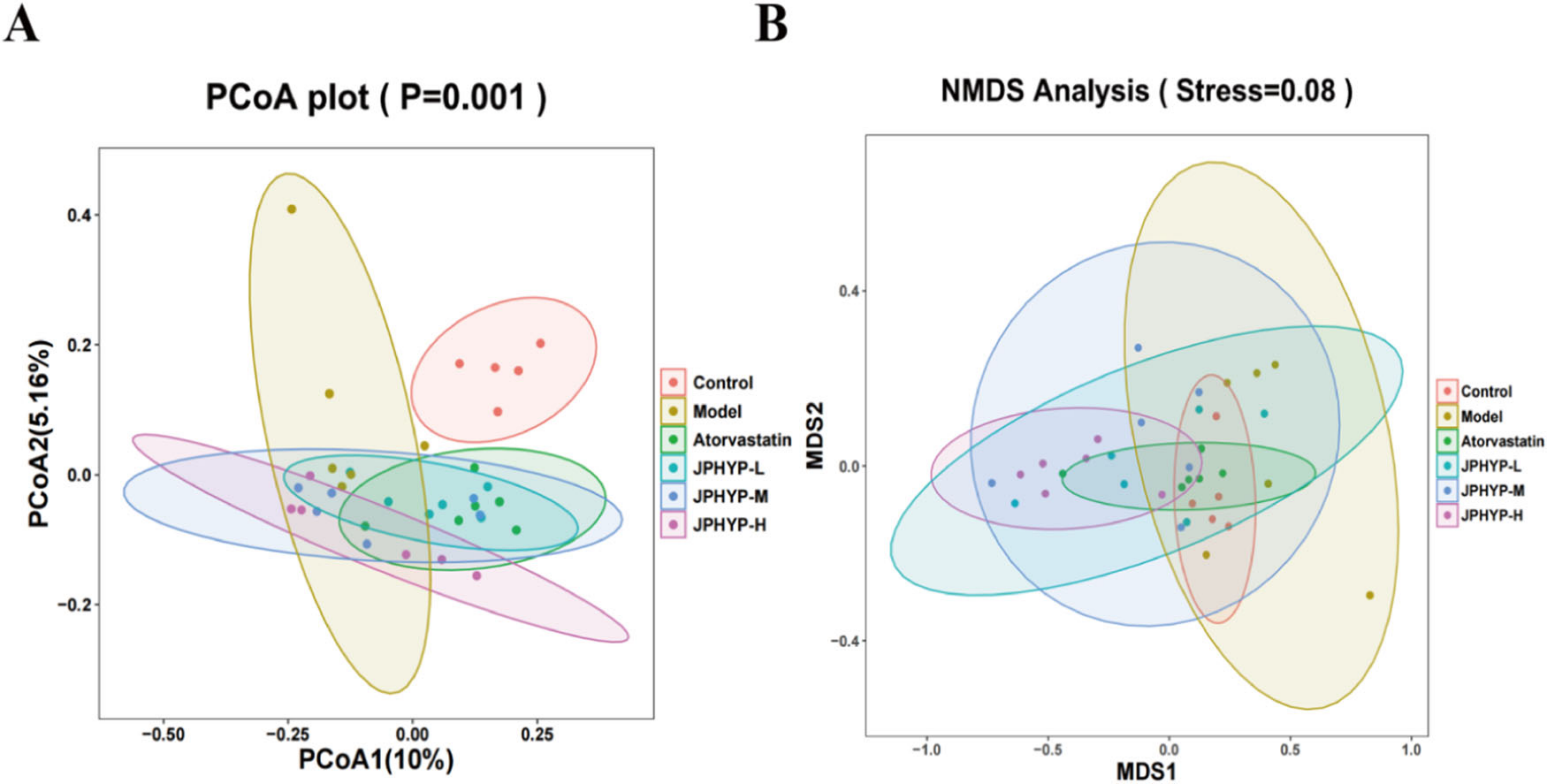
JPHYP restores beta diversity of intestinal microbiota in ApoE^−/−^ mice (*n* = 6). **A** PCoA analysis; **B** NMDS analysis

We used Illumina HiSeq 16S rRNA gene sequencing to examine the impact of JPHYP treatment on the composition of the gut microbiota. The results showed that 2523480 clean reads from 41 samples were obtained (Fig. 11A). As previously reported, three phyla—Bacteroidetes, Firmicutes, and Proteobacteria—accounted for roughly 93% of the gut bacterial community and dominated the composition at the phylum-level. The Venn diagram (Fig. 11B) summarizes the degrees of operational taxonomic units (OTUs) shared by the six groups. It reveals that 236 OTUs were shared by six groups, but some OTUs remained unique to each group (5274 for the control group, 2753 for the model group, 4944 for the atorvastatin group, 3333 for the JPHYP-L group, 3747 for the JPHYP-M group, and 4223 for the JPHYP-H group, respectively). This indicates that the JPHYP group had a higher OTU diversity. According to the Shannon index, JPHYP therapy under a high-fat diet significantly decreased the diversity of the gut microbiota. Furthermore, the JPHYP group’s alpha diversity is comparatively similar with that of the control group (Fig. 12). The microbiota composition of the high-fat diet group differed significantly from that of the control and JPHYP groups, which together make up 10% of the gut microbiota profile, according to principal coordinate analysis (PCoA) based on weighted UniFrac distance (Fig. 13A). Additionally, compared to the high-fat diet group, the gut microbial communities of the JPHYP group aggregated more closely to that of the control group, according to the weighted UniFrac NMDS (nonmetric multidimensional scaling) analysis (Fig. 13B).

**Fig. 14:**
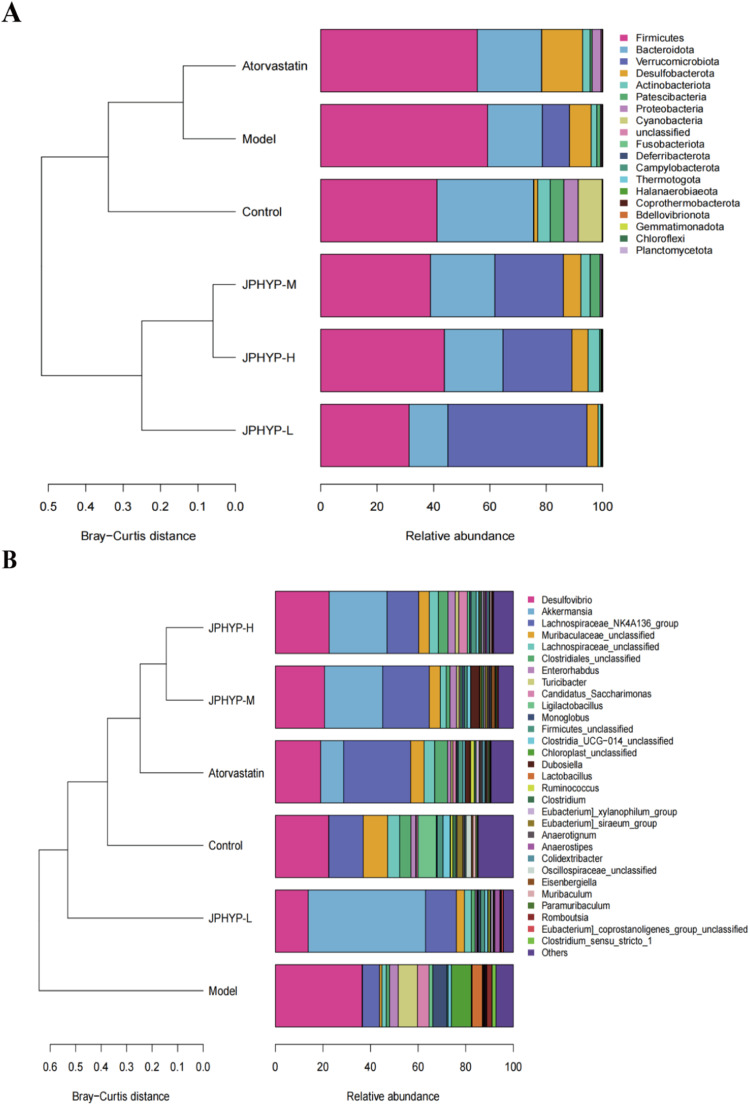
JPHYP improves the abnormal abundance of intestinal flora in ApoE^−/−^ mice (*n* = 6). **A** Phylum level species abundance histogram. **B** Genus level species abundance histogram

The classification results are compiled to determine the relative abundance of the samples at each level of phylum, order, family, genus, and species once the OTU annotation results are obtained. A distribution histogram for the community structure is created based on the findings. Dominant species are those that have a higher percentage in the histogram. According to the findings, *Firmicutes*, *Bacteroidetesother*, *Desulfobacterota*, *Verrucomicrobiota*, *Actinbacteria*, and *Proteobacteria* were found at the phylum level. The phylum comprises six primary groups of bacteria. Between the six groups, there were no appreciable variations in the quantity of *Bacteroidetes*. After JPHYP supplementation, the abundance of *Firmicutes* which is frequently elevated in the obese state was much lower than in the model group. When compared to the model group, the abundance of *Bacteroidetes* at the genus level rose considerably following JPHYP administration. The findings demonstrated that AS mice’s aberrant intestinal flora abundance could be corrected by JPHYP, and that HFD diet had altered the abundance of intestinal flora in AS mice (Fig. 14A, B).

**Fig. 15:**
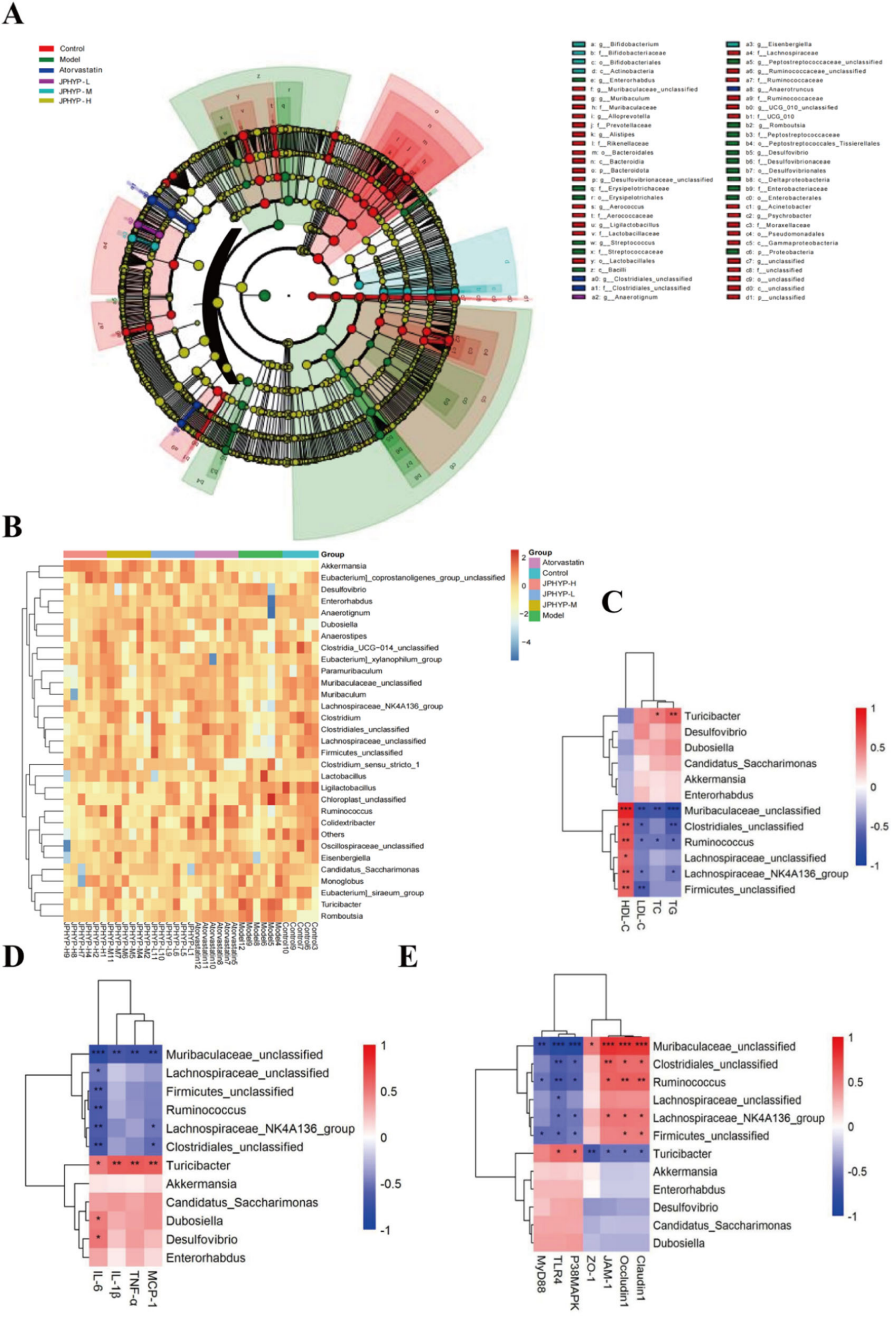
The impact of JPHYP on the gut microbiota composition. **A** Taxonomic cladogram generated by LEfSe analysis illustrating significant shifts in the gut microbiota in Control (red), Model (green), Atorvastatin (blue) or JPHYP (purple, sky blue and yellow) mice, respectively (*n* = 6). **B** Heat map comparison based on relative abundance of 30 key genera in each treatment group (*n* = 6). **C**–**E** Heat map describing the correlation of the abundances of key bacterial genera and AS parameters (*n* = 3). *p < 0.05, **p < 0.01, ***p < 0.001

The variations in intestinal flora species within each group are investigated using linear disciminant analysis Effect Size (LEfSe) analysis methods. The LEfSe analysis, which primarily examines the degree of contribution, annotations, and relative abundance of different species of different species, can be used to disclose the composition of different species of two or more biological groupings. The fan-shaped extension from the interior to the outside in the LEfSe analysis branch diagram indicates the classification levels from the door to belongings (or species). Important microorganisms in the control, model, and atorvastatin groups are represented by the red, green, and blue nodes, whereas the prominent microorganisms in the JPHYP group are represented by the purple, blue, and yellow nodes. As the figure illustrates, the model groups with differences are: *f - Desulfovibrionaceae*, *o - Desulfovibrionales*; abnormal species are: *f - Bifidobacteriaceae*, *o - Bifidobacteriales*, and *c - Actinobacteria*. The differences between the contributions of the control group are: *f - Prevotellaceae*, *o - Bacteroidales*, *c - Bacteroidia*, *f - Lactobacillaceae*). The findings demonstrate that JPHYP controls the stability of intestinal flora by boosting flora quantity (Fig. 15A).

Heat map analysis was used to look at the variations in the intestinal flora makeup of each experimental group. The depth of the filled color in the square represented the relative quantity of each bacteria in individual mouse. Following JPHYP treatment, the intestinal flora of AS mice varied in terms of the abundance of thirty distinct species. The relative abundances of *Bacteroidetes*, *Actinobacteriota*, and *Verrucomicrobiota* were all dramatically suppressed by the model group. The JPHYP treatment intervention resulted in a considerable increase in the abundance of the previously stated bacterial taxa. Conversely, the administration of JPHYP can considerably lessen the rise in *Firmicutes*, *Fusobacteriota*, and *Proteobacteria* brought on by a diet high in fat. When considered collectively, these findings suggest that JPHYP regulates the alterations in the intestinal flora of ApoE^−/−^ mice fed a high-fat diet, reducing the variations in intestinal flora between the JPHYP treatment group and the control group at various degrees. (Fig. 15B).

We used Spearman correlation analysis to investigate the relationship between gut microbiota and atherosclerosis markers. *Desulfovibrio* and *Firmicutes* are abundant in the model group; *Firmicute*s has a positive correlation with HDL-C levels and a negative correlation with LDL-C and IL-6 levels. It also has a negative correlation with the levels of TLR4, MyD88, and P38MAPK protein and a positive correlation with the intestinal tight junction proteins Occludin1 and Claudin1. *Turicibacter*, *Muribaculaceae_unclassified*, *Lachnospiraceae_NK4A136_group*, and *Lachnospiraceae_unclassified* are abundant in the drug administration group. *Turicibacter* has a positive correlation with TLR4 and P38MAPK pathway protein levels as well as positive correlations with TC, TG, TNF-α, IL-1β and MCP-1 levels. Associated, negatively linked with ZO-1, Occludin1, Claudin1, and JAM-1, intestinal tight junction proteins. Figure 15C–E shows that *Muribaculaceae_unclassified* has a substantial negative correlation with TLR4, MyD88, and P38MAPK protein levels, a positive correlation with HDL-C, a negative correlation with TC, TG, LDL-C, TNF-α, IL-1β, IL-6, and MCP-1 levels, and a negative correlation with TLR4.

## Discussion

This study demonstrated these main findings: (1) JPHYP contains ingredients that have the function of anti-atherosclerosis; (2) JPHYP alleviates AS in vivo and in vitro; (3) JPHYP can inhibit the level of LPS-induced inflammatory factors in RAW264.7 macrophages and reduce the expression of TLR4, MyD88, JNK, AP-1 and P38MAPK, which may be related to the regulation of TLR4/MyD88/MAPK signaling pathway; (4) JPHYP can improve blood lipids and inflammation in ApoE^−/−^ mice, reduce the generation of foam cells and plaques in aortic tissue, increase plaque stability, and regulate intestinal microbial imbalance, which may be related to the regulation of TLR4/MyD88/MAPK signaling pathway.

JPHYP is a traditional Chinese medicine component that is safe and effective. It is derived from the traditional Sijunzi Decoction. The entire formula eliminates blood stasis, phlegm, and strengthens the spleen while reviving qi. Poria cocos, which is good for the body and strengthens the spleen, fried Atractylodes macrocephala, and codonopsis pilosula are the ingredients in the prescription. Qi, in order for the blood vessels to remain clear and for the Qi and pulse to flow. Utilize ministers such as *tangerine peel*, *Pinellia ternata*, *Trichosanthes*, *Salvia miltiorrhiza*, *Gynostemma pentaphylla*, and *Alisma* to resolve phlegm, dry dampness, remove fat, and lessen turbidity. Use *raw hawthorn*, *safflower*, and *leeches* at the same time to help dissolve plaque, promote blood circulation, and eliminate blood stasis. Their demonstrated efficacy and wide range of application prospects have led to their widespread clinical use in AS. The first step in this study’s objective of achieving both qualitative and quantitative analysis is the identification of the sample’s chemical components using UPLC-Q-TOF/MS technology. It separates and identifies compounds using ultra-high performance liquid chromatography-mass spectrometry technology in conjunction with the annotation of mass spectrometry database information. Additionally, JPHYP provides classification, precise characterization, a thorough description of the material composition and content, and other data. The findings indicate that Salvianolic acid B, Gypenoside, Cryptotanshinone, Hesperidin, Nobiletin, and other substances are included in the material makeup of JPHYP. Yiqing [16] found that Salvianolic acid B has good anti-oxidative stress ability, which may Anti-AS by activating Nrf2 signaling pathway; Kaihua et al. [17] have shown that Cryptotanshinone is an effective drug in the treatment of cardiovascular diseases, and has the effects of anti-atherosclerosis, scavenging oxygen free radicals, improving hemodynamics, protecting myocardium and inhibiting myocardial apoptosis; Shafeeque et al. [18] have shown that Hesperidin reduces AS by improving insulin resistance and lipid distribution, inhibiting macrophage foam cell formation, antioxidant and anti-inflammation. Koga et al. [19] pointed out that Hesperidin inhibits the effect of varenicline on ApoE^−/−^. The aggravating effect of the entire aorta, aortic arch and aortic root in mice blocks AS plaque formation by down-regulating ox-LDL; Jinhong et al. [20] have shown that Nobiletin is the main component of the traditional Chinese medicine tangerine peel and has hypolipidemic, antioxidant and anti-inflammatory effects. These active compounds are present in JPHYP to treat and prevent AS. JPHYP dramatically decreased the quantities of inflammatory factors and ROS produced by macrophages in animal studies, and it enhanced the area of the aortic plaque, blood lipid levels, and inflammation in ApoE^−/−^ mice. These results align with the roles of chemical components in JPHYP and the outcomes of earlier studies. Furthermore, no discernible variation was observed in the survival rate of macrophages cultured with serum containing JPHYP when compared to the control group (Fig. 2E). In summary, we can say that JPHYP is secure from AS.

The human body’s “second genome” is frequently referred to as the gut microbiome. The gut microbiota and the incidence of CVD have been linked in numerous studies [21–23]. All bacteria have chromosomal genomes containing 16S rDNA [24], which is found on the tiny subunit of the ribosome in prokaryotic cells. Its mild structural and base organization complexity, along with its around 1540 bp length, make it simple to sequence, identify, analyze, and compare. In example, 16S rDNA homology analysis is useful for finding distant relationships and genera beyond the genus level. Identification of the microorganism species present in a sample can be achieved by comparing the 16S rDNA gene sequences from different organisms. *Bacteroidetes* and *Firmicutes* make up the majority of the two major phyla that make up the gut flora in humans—more than 90%. Other phyla that play less important but still important roles include *Actinobacteria*, *Cyanobacteria*, *Fusobacteria*, *Proteobacteria*, and *Verrucomicrobia*. The injection of JPHYP considerably improved the richness and diversity of intestinal flora in AS model mice, according to the results of the intestinal flora’s alpha diversity study. It has been proposed that JPHYP regulates intestinal flora variety in AS mice in a positive manner. The structural makeup of the intestinal flora following JPHYP intervention was comparable to that of the control group, according to the Beta diversity data, suggesting that JPHYP can restore the species diversity of the intestinal flora in AS mice. *Firmicutes* is the leading species in the model group, according to the examination of the differences and composition of the gut flora. According to reports, AS may result from an accumulation of inflammation brought on by the relative abundance of *Firmicutes* [25]. The study’s findings demonstrated that *Bacteroidetes* dominated the bacteria in the group that received JPHYP treatment, suggesting that JPHYP can boost *Bacteroidetes*’ abundance. A class of bacteria known as *Bacterialoides* that produce short-chain fatty acids aids in the fight against obesity brought on by a high-fat diet. *Bacteroidetes* are the bacteria that have a higher relative abundance at the genus level in the control group. Mice in the control group are more hospitable to *Bacteroidetes*, as evidenced by studies demonstrating that this type of bacteria can lower intestinal inflammation in mice [26], colonization with good bacteria. *Firmicutes* is the genus-level bacteria with the highest relative abundance in the model group. *Firmicutes* have been shown to induce intestinal barrier disruption, weight gain, and colon inflammation in mice [27], suggesting that the intestinal environment of the model group of mice may not be conducive to the growth of beneficial bacteria, colonization. *Muribaculaceae* is the genus with a higher relative abundance following JPHYP administration intervention. This bacterial genus is a recently formed one. The S24-7 family was the previous name for it. It is a member of the phylum *Bacteroidetes* and is found in high concentration in the intestines of mice. Intestinal homeostasis can be maintained through competition, production of antimicrobial substances and regulation of the immune system. Changes in *Muribaculaceae* are mainly related to various dietary treatments, host conditions or rodent colonization processes, but the specific characteristics of this bacterial genus Function currently lacks in-depth research [28–30]. The heat map analysis results showed that JPHYP intervention could promote the increase in the level of *Bacteroidetes* in the intestinal flora of AS mice. At the same time, according to Spearman correlation analysis, *Firmicutes* was positively correlated with HDL-C levels, negatively correlated with LDL-C and IL-6 levels, positively correlated with HDL-C levels, and negatively correlated with TLR4, MyD88 and P38MAPK protein levels, positively correlated with intestinal tight junction proteins Occludin1 and Claudin1. And some studies have shown that the increase in *Firmicutes* will lead to metabolic syndromes such as obesity and diabetes in humans. In the administration group, *Turicibacter* was positively correlated with TC, TG, TNF-α, IL-1β, MCP-1, CXCL1 and IFN-β levels, with TLR4 and P38MAPK protein levels, and with intestinal tight junction proteins ZO-1, Occludin1, Claudin1 and JAM-1 were negatively correlated. *Muribaculaceae* was positively correlated with HDL-C, negatively correlated with the levels of TC, TG, LDL-C, TNF-α, IL-1β, IL-6 and MCP-1, and negatively correlated with the pathway TLR4, MyD88 and P38MAPK proteins. The levels were significantly negatively correlated with intestinal tight junction proteins ZO-1, Occludin1, Claudin1 and JAM-1. Therefore, we speculate that JPHYP intervention may exert an anti-AS effect by increasing the abundance of the genus *Turicibacter* and *Muribaculaceae*. The above results show that the structure of the intestinal flora in the model group is disordered and the abundance of harmful bacteria increases, while JPHYP effectively improves the imbalance of intestinal flora, reduces the abundance of harmful bacteria and increases the abundance of beneficial bacteria, inhibiting the occurrence of inflammation in the intestines.

An increase in pathogenic bacteria and disruption of the intestinal flora can result in endotoxemia, damage the intestinal mucosal barrier, alter intestinal permeability, alter intestinal flora, and allow a significant amount of lipopolysaccharide (LPS) to enter the bloodstream, which can trigger a systemic inflammatory response. Foam cell development will be accelerated by this increase in LPS since it will also upregulate the expression of vascular chemokines and cell adhesion molecules [31]. Numerous goblet cells, which release mucin and create a thick mucus-protective layer of intestinal epithelial cells, are seen in significant numbers in the mucosal layer of the large intestine [32]. Because of tight connections between epithelial cells, pathogens cannot penetrate gut tissue. Typically, protein complexes like ZO-1, Occludin1, Claudin1, and JAM-1 make up the junction structure. In cases of metabolic disorders, the intestinal barrier’s integrity is crucial. Research has indicated that disruption to the intestinal barrier may increase the risk of AS [33]. The intestinal flora structure of the organism is stable and kept in a state of relative equilibrium under normal conditions. However, the intestinal mucosal barrier may become damaged due to pathological conditions like intestinal bacterial overgrowth or inflammatory stimulation. This can lead to increased intestinal permeability and the passage of pathogenic products through the damaged intestinal mucosal barrier. A number of illnesses are brought on by the portal vein entering the bloodstream. According to this study, the model group of mice’s large intestine’s muscularis propria thinned, and goblet cells and the intestinal mucus layer suffered injury, which reduced the amount of intestinal mucin secreted. Mice’s intestinal barrier damage and mucin secretion were repaired during JPHYP treatment. reduction of secretion. Furthermore, the LPS level is markedly elevated in AS model mice, suggesting a disruption in the function of the intestinal mucosal barrier. Through the compromised intestinal mucosal barrier, a significant quantity of LPS can enter the bloodstream, potentially causing persistent inflammation and encouraging the development of AS plaques. During development, there was a notable down-regulation of the tight junction protein expression levels in large intestine tissue for ZO-1, Occludin1, Claudin1, and JAM-1, which resulted in a rise in the permeability of intestinal epithelial tissue. Following JPHYP delivery, there was a notable decrease in the level of LPS. decreased and markedly reduced the expression of ZO-1, Occludin1, Claudin1, and JAM-1 proteins. As a result, JPHYP can lessen LPS concentration, preserve the integrity of the intestinal tissue structure, and enhance the state of intestinal barrier damage in AS model mice. In conclusion, JPHYP protects the intestinal barrier of AS model mice, enhances the diversity and richness of the intestinal flora, and somewhat lowers the production of LPS. As a result, JPHYP may balance the unbalanced gut flora in ApoE^−/−^ mice, enhance the function of the intestinal mucosal barrier, and decrease the amount of LPS that enters the bloodstream. These actions will inhibit the TLR4/MyD88/MAPK inflammatory signaling pathway, downregulate the expression of inflammatory factors, and ultimately alleviate AS.

Both systemic inflammation and gut microbiota can influence lipid metabolism and serve as environmental factors that trigger the development of metabolic and cardiovascular diseases. Inflammation plays an important role in the pathogenesis of AS. Different intestinal flora may have preventive and anti-atherosclerotic effects. More and more studies have confirmed that inflammation and intestinal flora are closely related to AS [34–36], but the specific mechanism is still unclear. The TLR4/MyD88/MAPK pathway is an important signaling pathway that mediates inflammatory responses in the body. TLR4 is a highly conserved transmembrane receptor and an important mediator of the innate immune system. It regulates the activity of monocyte macrophages. TLR4 can recognize specific ligands and activate inflammatory responses to promote the formation of AS. It is a key regulator of the occurrence and development of AS [37]. Studies have shown that vascular endothelial cells and macrophages in AS lesions can express TLR4 receptors, and the TLR4 signaling pathway relies on MyD88 to induce and activate related signaling factors, such as NF-κB, IL-1 and IL-6, thereby causing an inflammatory response. and regulate the transcription of cell-related genes. As a transduction protein, MyD88 plays an important role in the TLR4 signaling pathway. The signaling pathways it relies on and the gene products it regulates play a key role in both innate immunity and adaptive immunity. It can activate the MAPK pathway and its important branch c-Jun N-terminal kinase (JNK) signaling pathway activates activated protein 1 (AP-1), induces the expression of related inflammatory cytokines, mediates the transcription of related target genes, and then causes the body’s inflammatory and immune responses [38]. MAPK is a serine/threonine protein 57 kinase family. MAPK activation induces the expression of pro-inflammatory cytokines and is a key regulator of AS. Multiple studies have shown that repressing the MAPK pathway can reduce inflammation and AS [39]. The results showed that TLR4, MyD88, JNK, AP-1 and P38MAPK were significantly increased in the model group, and JPHYP intervention reversed the increase of these five proteins. Previous studies have reported that TLR4 deletion alone can lead to a significant reduction in aortic lesion size [40–42]. In this study, we found that JPHYP may antagonize AS by regulating the MAPK signaling cascade initiated by TLR4.

In summary, our research offers mounting proof that JPHYP mitigates AS brought on by a high-fat diet. In ApoE^−/−^ mice, JPHYP can control blood lipid levels and the makeup of the intestinal flora, improve intestinal barrier function, lower the expression of LPS, and lessen inflammation. It’s possible that the method involves blocking the TLR4/MyD88/MAPK signaling pathway. These results lend more credence to the theory that changes in the gut microbiota’s composition are closely related to the mechanism behind JPHYP’s anti-atherosclerotic actions. This is the first study to demonstrate how JPHYP treatment specifically alters the abundance of *Turicibacter*, *Muribaculaceae*, and *Bacteroidetes*. This finding supports the idea that drugs that manipulate the gut microbiota could be used to treat AS and provides a solid scientific foundation for future research and clinical applications.

## References

[1] Goldsborough, 3rd, E., Tasdighi, E., & Blaha, M. J. (2023). Assessment of cardiovascular disease risk: a 2023 update. *Current Opinion in Lipidology*, *34*(4), 162–173. 37431303 10.1097/MOL.0000000000000887

[2] Shi, T., Liu, K. & Peng, Y. et al. (2023). Research progress on the therapeutic effects of nanoparticles loaded with drugs against atherosclerosis. *Cardiovascular Drugs and Therapy*, *15*(3), 638–639. 10.1007/s10557-023-07461-0 37178241

[3] Li, Y., Zhang, Y., & Lu, J. et al. (2021). Anti-inflammatory mechanisms and research progress of colchicine in atherosclerotic therapy. *Journal of Cellular and Molecular Medicine*, *25*(17), 8087–8094. 34312998 10.1111/jcmm.16798 PMC8419170

[4] Zhang, F., & Qiao, S. (2022). Research Progress on the Relationship Between Inflammation and Colorectal Cancer. *Annals of Gastroenterological Surgery*, *6*(2), 204–211. 35261946 10.1002/ags3.12517 PMC8889855

[5] Capodanno, D., & Angiolillo, D. J. (2018). Canakinumab for secondary prevention of atherosclerotic disease. *Expert Opinion on Biological Therapy*, *18*(2), 215–220. 29265905 10.1080/14712598.2018.1420776

[6] Kofoed, K. F., Engstrøm, T., & Sigvardsen, P. E. et al. (2021). Prognostic Value of Coronary CT Angiography in Patients With Non-ST-Segment Elevation Acute Coronary Syndromes. *Journal of the American College of Cardiology*, *77*(8), 1044–1052. 33632478 10.1016/j.jacc.2020.12.037

[7] Yoshida, N., Emoto, T., & Yamashita, T. et al. (2018). Bacteroides vulgatus and Bacteroides dorei Reduce Gut Microbial Lipopolysaccharide Production and Inhibit Atherosclerosis. *Circulation*, *138*(22), 2486–2498. 30571343 10.1161/CIRCULATIONAHA.118.033714

[8] Wu, T., Yu, Q., Luo, Y., et al. (2023). Whole-Grain Highland Barley Attenuates Atherosclerosis Associated with NLRP3 Inflammasome Pathway and Gut Microbiota in ApoE Mice. *Nutrients*, *15*(19), 4186. 10.3390/nu15194186 PMC10574078 37836470

[9] Li, R. J., Jie, Z. Y., & Feng, Q. et al. (2021). Network of Interactions Between Gut Microbiome, Host Biomarkers, and Urine Metabolome in Carotid Atherosclerosis. *Frontiers in Cellular and Infection Microbiology*, *11*, 708088. 34692558 10.3389/fcimb.2021.708088 PMC8529068

[10] Wei, R., Lv, X., & Fang, C. et al. (2022). Silencing TUFM Inhibits Development of Monocrotaline-Induced Pulmonary Hypertension by Regulating Mitochondrial Autophagy via AMPK/mTOR Signal Pathway. *Oxidative Medicine and Cellular Longevity*, *2022*, 4931611. 35936222 10.1155/2022/4931611 PMC9348918

[11] Dong, Y., Cheng, H., & Liu, Y. et al. (2019). Red yeast rice ameliorates high-fat diet-induced atherosclerosis in Apoe mice in association with improved inflammation and altered gut microbiota composition. *Food & Function*, *10*(7), 3880–3889. 31187839 10.1039/c9fo00583h

[12] Zhi, W., Liu, Y., & Wang, X. et al. (2023). Recent advances of traditional Chinese medicine for the prevention and treatment of atherosclerosis. *Journal of Ethnopharmacology*, *301*, 115749. 36181983 10.1016/j.jep.2022.115749

[13] Zhang, X. M., Gan, X., & Zhang, H. X. et al. (2023). Treatment of atherosclerosis from “spleen-phlegm-stasis”[J]. *Corps Medicine*, *21*(01), 59–61.

[14] Zhang, X. M., Ye, D., & Zhang, H. X. et al. (2015). Treatment of 68 cases of hyperlipidemia with ShenlingBaizhu powder. *Chinese Journal of Experimental Formulae*, *21*(12), 158–161.

[15] Qiao, H. & Zhang, X. (2023). Clinical observation and network pharmacology study of Jianpi Huayu Recipe against carotid atherosclerosis. *Liaoning traditional Chinese medicine*, *33*(17), 4261.

[16] Wang. Y. (2022). *Study on the anti-oxidative stress effect of stachydrine based on network pharmacology*. Jilin University.

[17] L, K. H., L, F., & Z, H. et al. (2022). Simultaneous determination of the contents of 10 ingredients in Guanxinshutong Capsules using the one-measurement multiple-evaluation method. *Journal of Pharmaceutical Sciences*, *57*(06), 1880–1886.

[18] Sun, Y. Z., Chen, J. F., & Shen, L. M. et al. (2017). Anti-atherosclerotic effect of hesperidin in LDLr mice and its possible mechanism. *European Journal of Pharmacology*, *815*, 109–117. 28899694 10.1016/j.ejphar.2017.09.010

[19] Koga, M., Kanaoka, Y., & Inada, K. et al. (2020). Hesperidin blocks varenicline-aggravated atherosclerotic plaque formation in apolipoprotein E knockout mice by downregulating net uptake of oxidized low-density lipoprotein in macrophages. *Journal of Pharmacological Sciences*, *143*(2), 106–111. 32173266 10.1016/j.jphs.2020.01.012

[20] W, J. H., W, J. S., & L, Q. et al. (2019). Study on coagulation, hemostasis and antithrombotic effects of tangerine peel. *China Wild Plant Resources*, *38*(06), 33–37.

[21] Ou, Y., Zhang, C., & Yao, M., et al. (2019). Gut Flora: Novel Therapeutic Target of Chinese Medicine for the Treatment of Cardiovascular Diseases. *Evidence-Based Complementary and Alternative Medicine : eCAM*, *2019*, 3719596. 31531111 10.1155/2019/3719596 PMC6721502

[22] Zheng, W. C., Chan, W., & Dart, A. et al. (2024). Novel therapeutic targets and emerging treatments for atherosclerotic cardiovascular disease. *European Heart Journal Cardiovascular Pharmacotherapy*, *10*(1), 53–67. 37813820 10.1093/ehjcvp/pvad074

[23] Ganesh, N., van der Vorst, E. P. C., & Spiesshöfer, J. et al. (2022). Gut immune cells-A novel therapeutical target for cardiovascular disease?. *Frontiers in Cardiovascular Medicine*, *9*, 943214. 36046186 10.3389/fcvm.2022.943214 PMC9421162

[24] Yang, Z., Lin, S., & Liu, Y. et al. (2023). Targeting intestinal microecology: potential intervention strategies of traditional Chinese medicine for managing hypertension. *Frontiers in Pharmacology*, *14*, 1171119. 37324472 10.3389/fphar.2023.1171119 PMC10264781

[25] Song, X., Zhong, L., & Lyu, N. et al. (2019). Inulin Can Alleviate Metabolism Disorders in ob/ob Mice by Partially Restoring Leptin-related Pathways Mediated by Gut Microbiota. *Genomics, Proteomics & Bioinformatics*, *17*(1), 64–75. 10.1016/j.gpb.2019.03.001 PMC6520907 31026583

[26] Gao, M., Heng, X., Jin, J. et al. (2022). Gypenoside XLIX Ameliorate High-Fat Diet-Induced Atherosclerosis via Regulating Intestinal Microbiota, Alleviating Inflammatory Response and Restraining Oxidative Stress in ApoE Mice. *Pharmaceuticals*, *15*(9), 1056. 10.3390/ph15091056 PMC9501270 36145277

[27] Hao, H., Li, Z., & Qiao, S. Y., et al. (2023). Empagliflozin ameliorates atherosclerosis via regulating the intestinal flora. *Atherosclerosis*, *371*, 32–40. 36990029 10.1016/j.atherosclerosis.2023.03.011

[28] Lagkouvardos, I., Lesker, T. R., Hitch, T. C. A. et al. (2019). Sequence and cultivation study of Muribaculaceae reveals novel species, host preference, and functional potential of this yet undescribed family. *Microbiome*, *7*, 28. 10.1186/s40168-019-0637-2 PMC6381624 30782206

[29] Tropini, C., Moss, E. L., Merrill, B. D. et al. (2018). Transient Osmotic Perturbation Causes Long-Term Alteration to the Gut Microbiota. *Cell*, *173*, 1742–1754.e17. 10.1016/j.cell.2018.05.008 PMC6061967 29906449

[30] Shen, T. C., Chehoud, C., Ni, J., et al. (2016). Dietary Regulation of the Gut Microbiota Engineered by a Minimal Defined Bacterial Consortium. *PLoS One*, *11*, e0155620. 10.1371/journal.pone.0155620 PMC4866709 27176607

[31] Meng, Q., Pu, L., & Lu, Q. et al. (2021). Morin hydrate inhibits atherosclerosis and LPS-induced endothelial cells inflammatory responses by modulating the NFκB signaling-mediated autophagy. *International Immunopharmacology*, *100*, 108096. 34464886 10.1016/j.intimp.2021.108096

[32] Yang, S., & Yu, M. (2021). Role of Goblet Cells in Intestinal Barrier and Mucosal Immunity. *Journal of Inflammation Research*, *14*, 3171–3183. 34285541 10.2147/JIR.S318327 PMC8286120

[33] Liu, F., Shan, S., & Li, H. et al. (2021). Millet shell polyphenols prevent atherosclerosis by protecting the gut barrier and remodeling the gut microbiota in ApoE mice. *Food & Function*, *12*(16), 7298–7309. 34169953 10.1039/d1fo00991e

[34] Brandsma, E., Kloosterhuis, N. J., & Koster, M. et al. (2019). A Proinflammatory Gut Microbiota Increases Systemic Inflammation and Accelerates Atherosclerosis. *Circulation Research*, *124*(1), 94–100. 30582442 10.1161/CIRCRESAHA.118.313234 PMC6325767

[35] Deng, B., Tao, L., & Wang, Y. (2022). Natural products against inflammation and atherosclerosis: Targeting on gut microbiota. *Frontiers in Microbiology*, *13*, 997056. 36532443 10.3389/fmicb.2022.997056 PMC9751351

[36] Li, F., Zhang, T., & He, Y. et al. (2020). Inflammation inhibition and gut microbiota regulation by TSG to combat atherosclerosis in ApoE mice. *Journal of Ethnopharmacology*, *247*, 112232. 31606534 10.1016/j.jep.2019.112232

[37] Hayashi, C., Papadopoulos, G., & Gudino, C. V. et al. (2012). Protective role for TLR4 signaling in atherosclerosis progression as revealed by infection with a common oral pathogen. *Journal of Immunology*, *189*(7), 3681–3688. 10.4049/jimmunol.1201541 PMC3448820 22956579

[38] Lupi, L. A., Cucielo, M. S., & Silveira, H. S. et al. (2020). The role of Toll-like receptor 4 signaling pathway in ovarian, cervical, and endometrial cancers. *Life Sciences*, *247*, 117435. 32081661 10.1016/j.lfs.2020.117435

[39] Feng, M., Kong, S. Z., & Wang, Z. X. et al. (2017). The protective effect of coptisine on experimental atherosclerosis ApoE mice is mediated by MAPK/NF-κB-dependent pathway. *Biomedicine & Pharmacotherapy Biomedecine & Pharmacotherapie*, *93*, 721–729. 28700976 10.1016/j.biopha.2017.07.002

[40] Liu, P., Huang, G. & Cao, Z. et al. (2017). Haematopoietic TLR4 deletion attenuates perivascular brown adipose tissue inflammation in atherosclerotic mice. Biochimica et biophysica acta. *Molecular and Cell Biology of Lipids*, *1862*(9), 946–957. 28579235 10.1016/j.bbalip.2017.05.012

[41] Doddapattar, P., Jain, M., & Dhanesha, N. et al. (2018). Fibronectin Containing Extra Domain A Induces Plaque Destabilization in the Innominate Artery of Aged Apolipoprotein E-Deficient Mice. *Arteriosclerosis, Thrombosis, and Vascular Biology*, *38*(3), 500–508. 29326316 10.1161/ATVBAHA.117.310345 PMC5823768

[42] Chen, J., Tang, Z., & Chen, Z. et al. (2023). MicroRNA-218-5p regulates inflammation response via targeting TLR4 in atherosclerosis. *BMC Cardiovascular Disorders*, *23*(1), 122. 36890438 10.1186/s12872-023-03124-y PMC9996974

